# Correction: LprG-Mediated Surface Expression of Lipoarabinomannan Is Essential for Virulence of *Mycobacterium tuberculosis*

**DOI:** 10.1371/journal.ppat.1004489

**Published:** 2014-10-06

**Authors:** The PLOS Pathogens Staff

## Notice of Republication

This article was republished on September 24, 2014 to correct errors in the Competing Interests Statement. Please download this article again to view the correct version. The corrected article is provided here for reference.

## Supporting Information

File S1 Republished corrected article.
(PDF)
