# Correction: LprG-Mediated Surface Expression of Lipoarabinomannan Is Essential for Virulence of *Mycobacterium tuberculosis*

**DOI:** 10.1371/journal.ppat.1005336

**Published:** 2015-12-09

**Authors:** Rajiv L. Gaur, Kangning Ren, Antje Blumenthal, Suresh Bhamidi, Sara Gibbs, Mary Jackson, Richard N. Zare, Sabine Ehrt, Joel D. Ernst, Niaz Banaei

There is an error in Fig 1C. Lanes shown formed part of a larger gel, which is included in this Correction as S1 Fig.

**Fig 1:**
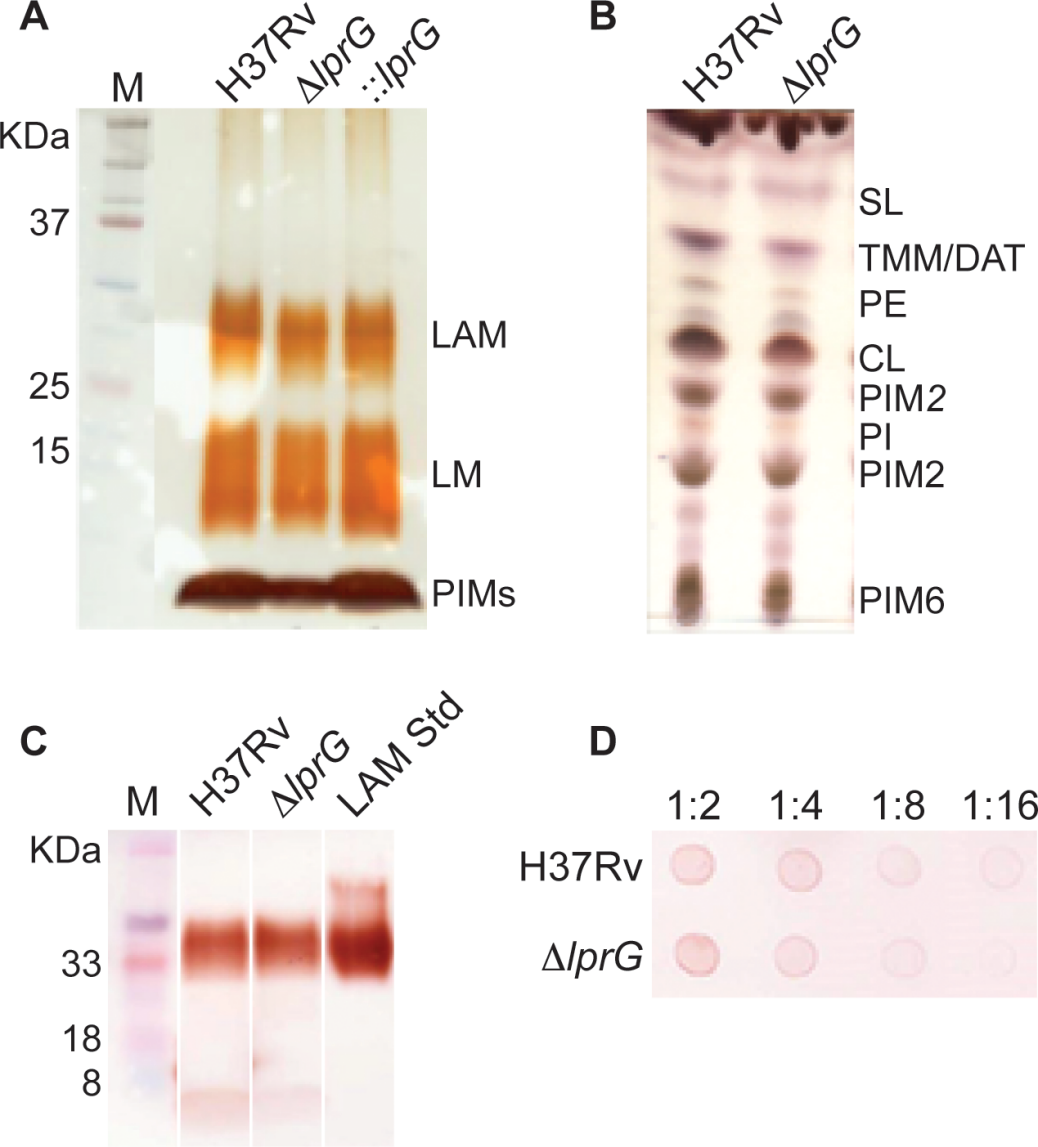
The *lprG* mutant has normal LAM content in the cell envelope. (A) SDS/PAGE analysis of phosphatidylinositol mannosides (PIMs), lipomannan (LM) and LAM prepared from wild-type (H37Rv), *lprG* mutant (Δ*lprG*), and Δ*lprG* complemented with *lprG*-Rv1410c (::*lprG*). LM and LAM extracted from equal amounts of bacterial cells were separated on a 10–20% Tricine gel and visualized by periodic acid/Schiff reagent staining. (B) Thin-layer chromatograms of total lipids extracted from H37Rv and Δ*lprG*. The same amounts of total lipids extract from bacilli grown in GAS medium were loaded for each strain. Thin-layer chromatogram plates were run in the solvent system CHCl_3_/CH_3_OH/H_2_O (65:25:4, by vol.) and revealed with α-naphthol. SL, sulfolipid; TMM, trehalose monomycolates; DAT, diacyltrehaloses; PE, phosphatidylethanolamine; CL, cardiolipin; PIM_2_, phosphatidylinositol dimannoside; PI, phosphatidylinositol; PIM_6_, phosphatidylinositol hexamannosides. (C) SDS/PAGE immunoblot for LAM analysis in H37Rv and Δ*lprG* cellular extracts. Extracts normalized to protein concentration were separated on a 15% SDS/PAGE gel and transferred to PVDF membrane. The blot was blocked, and then stained with anti-LAM pAb (α-LAM) followed by goat anti-rabbit IgG-HRP secondary antibody. The blot was washed and imaged after adding 30% 3,3’-diaminobenzidine tetrahydrochloride solution plus 0.0005% H_2_O_2_. LAM Std, purified H37Rv LAM standard. Lanes shown formed part of a larger gel. (D) Spot immunoblot for analysis of capsular α-glucan. Capsular content extracted from equal numbers of bacteria were spotted on PVDF membrane and stained with goat anti-phosphatidylinositol-glycans pAb followed by donkey anti-goat IgG-HRP secondary antibody. The membrane was developed and imaged as described in C. Dilutions of extract spotted on membrane are shown. Data is representative of two independent experiments.

The corrected version of Fig 1 can be seen here.

## Supporting Information

S1 Fig The original SDS/PAGE immunoblot for LAM analysis in H37Rv and Δ*lprG* cellular extracts. Extracts normalized to protein concentration were separated on a 15% SDS/PAGE gel and transferred to PVDF membrane. The blot was blocked, and then stained with anti-LAM pAb (α-LAM) followed by goat anti-rabbit IgG-HRP secondary antibody. The blot was washed and imaged after adding 30% 3,3’-diaminobenzidine tetrahydrochloride solution plus 0.0005% H_2_O_2_. LAM Std, purified H37Rv LAM standard. Soluble cell wall, purified H37Rv cell wall extract.
(PPTX)
