# Supplementary figures and images for: LprG-Mediated Surface Expression of Lipoarabinomannan Is Essential for Virulence of Mycobacterium tuberculosis
Source: PLoS Pathog. 2014 Sep 18;10(9):e1004376. doi: 10.1371/journal.ppat.1004376 (PMC4169494; doi:10.1371/journal.ppat.1004376)

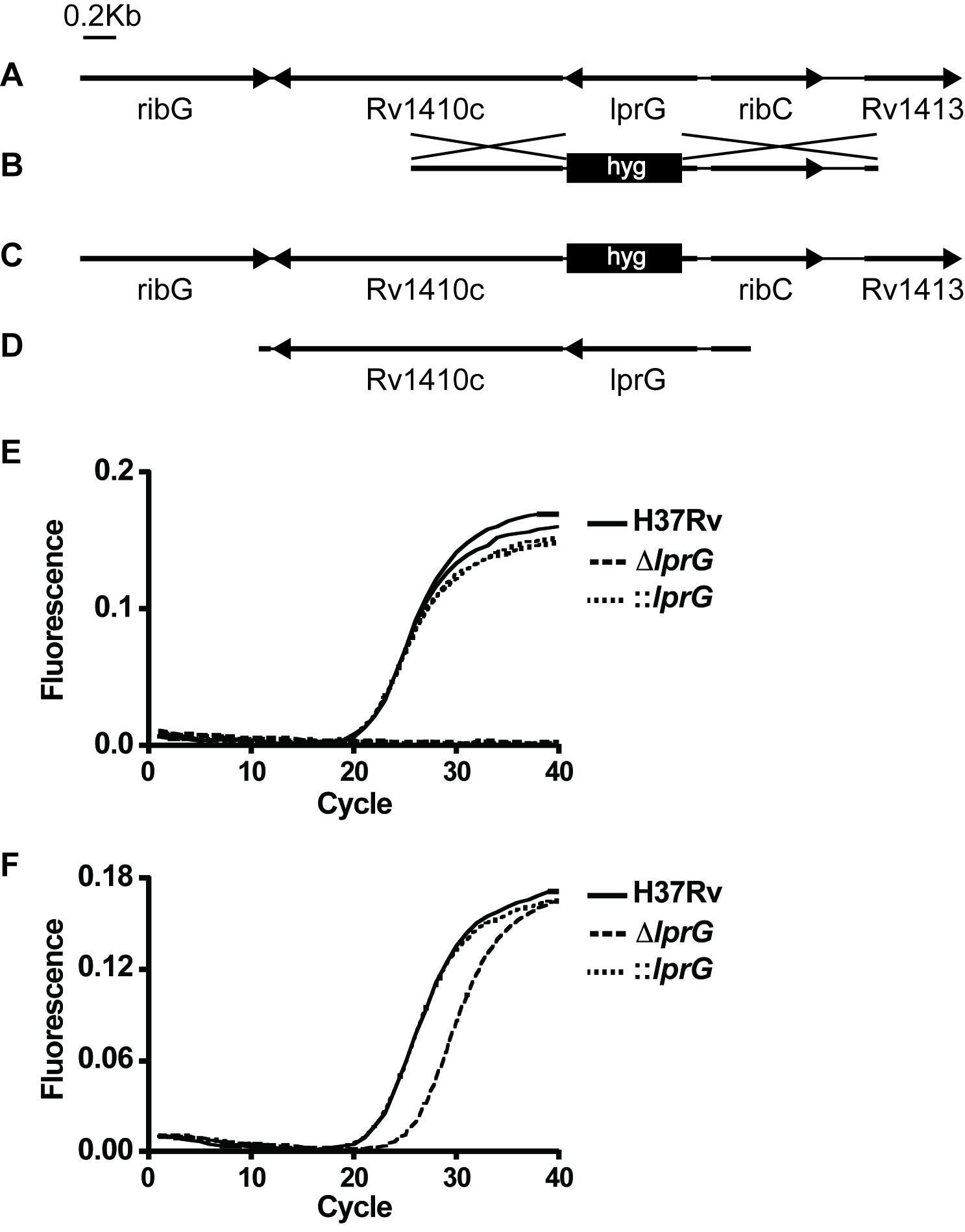

Supplement: Figure S1 — Construction of the lprG mutant. (A) The genomic map of lprG-Rv1410c operon in M. tuberculosis H37Rv. (B) The allelic exchange substrate used to construct the lprG mutant (ΔlprG). Hyg, hygromycin. (C) The genomic map of lprG-Rv1410c operon in ΔlprG. The black box represents region deleted from lprG. (D) The lprG-Rv1410c genomic fragment used to complement ΔlprG. (E) Real-time PCR amplification plot showing amplification of lprG from genomic DNA. (F) Reverse-transcriptase real-time PCR amplification plot showing amplification of Rv1410c from cDNA. (TIF) [file ppat.1004376.s001.tif]

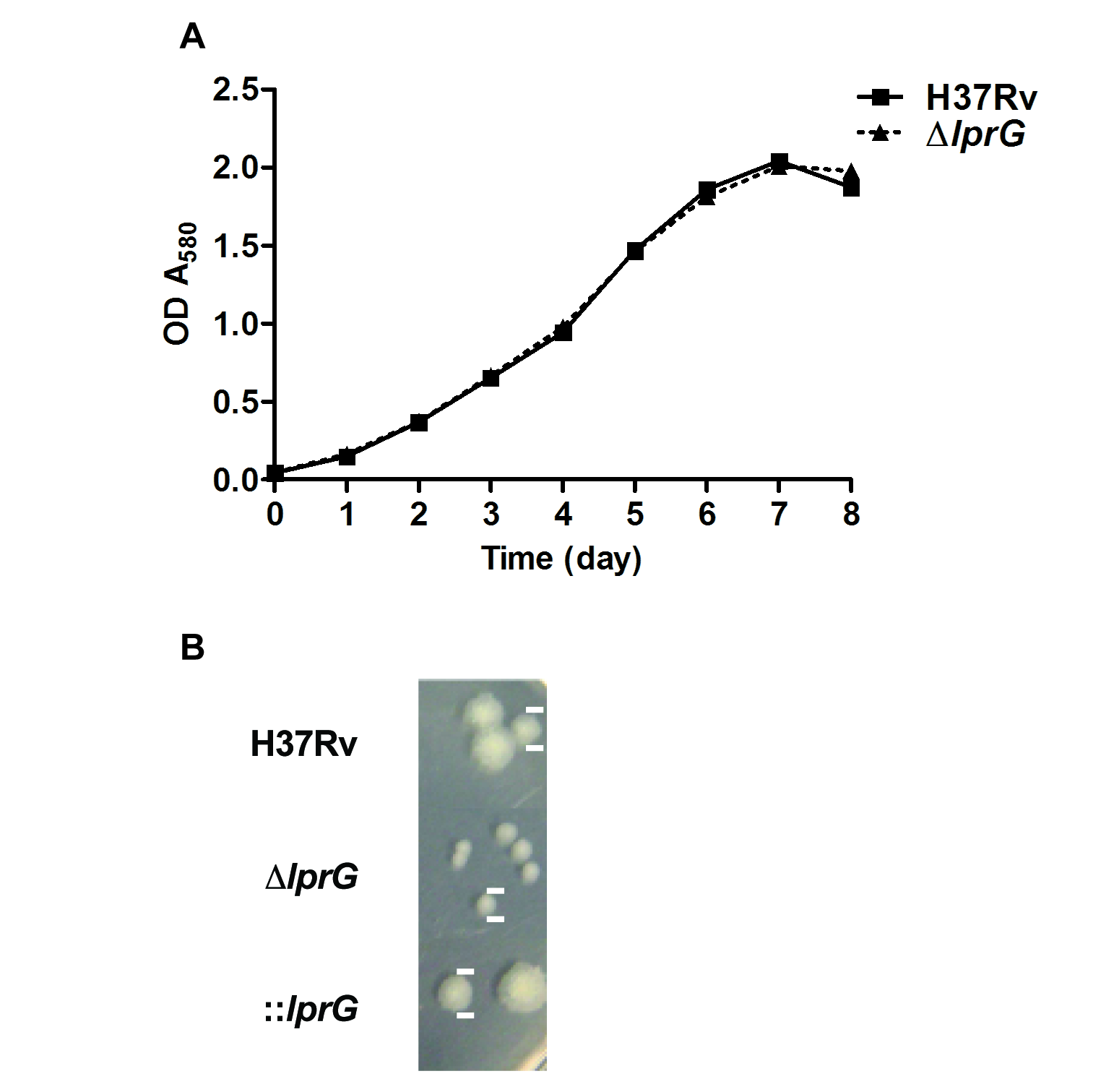

Supplement: Figure S2 — The lprG mutant has normal growth kinetics in broth. Mid-log shaking broth cultures of wild-type (H37Rv), lprG mutant (ΔlprG), and ΔlprG complemented with lprG-Rv1410c (::lprG) were diluted to ODA580 of 0.05 in Middlebrook 7H9 broth with Tween-80. (A) The optical density of shaking cultures was measured daily. (B) Images of colonies grown on Middlebrook 7H9 agar for 3 wk. White bars mark the borders of colonies. (TIF) [file ppat.1004376.s002.tif]

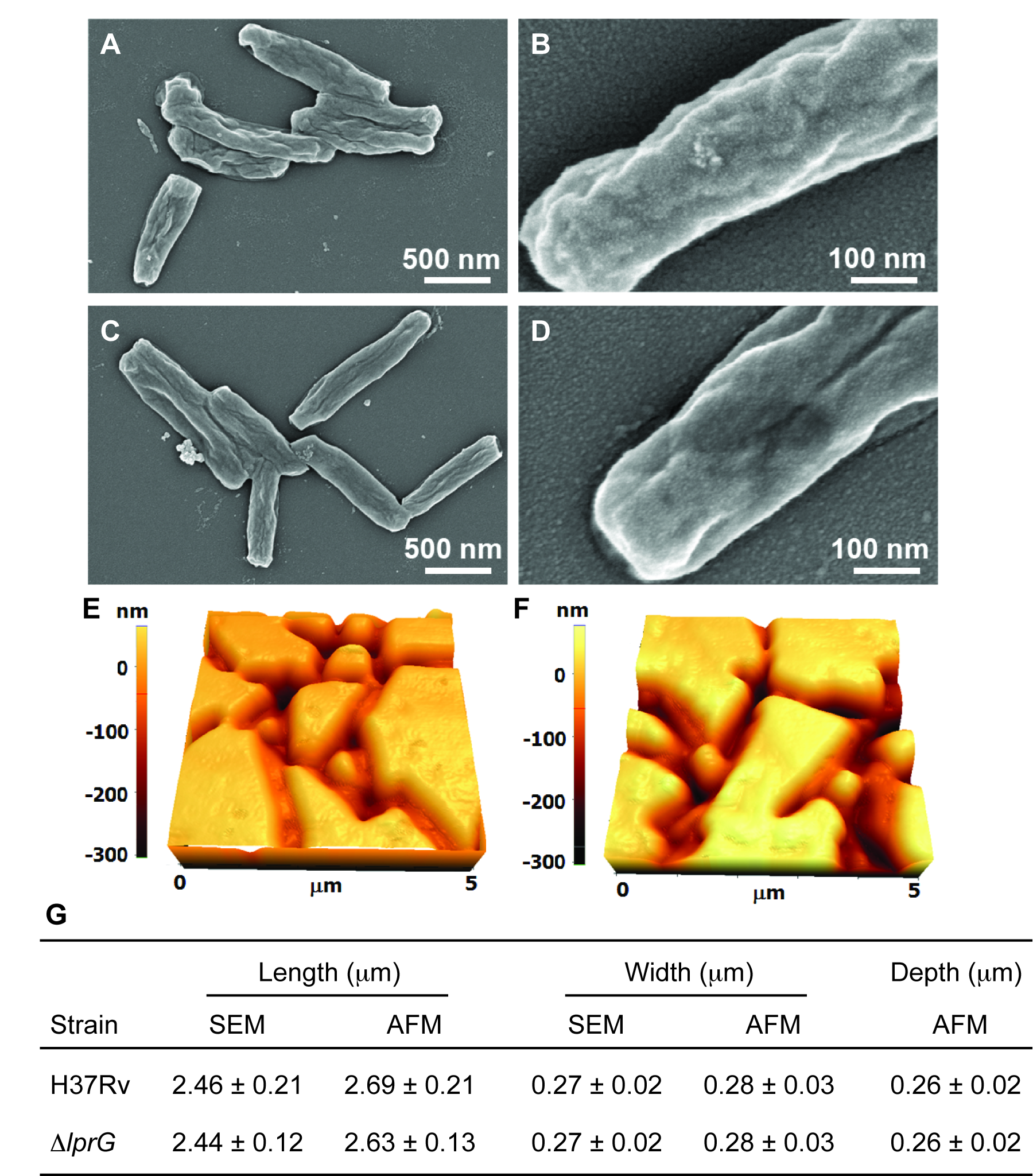

Supplement: Figure S3 — The lprG mutant has normal cellular dimensions. SEM micrographs of H37Rv (A and B) and ΔlprG (C and D). Wild-type (H37Rv) and lprG mutant (ΔlprG) bacilli grown in Middlebrook 7H9 broth were fixed with 4% formaldehyde, 2% glutaraldehyde, followed by 1% aqueous osmium tetroxide, and then dehydrated and coated with a thin layer of Au/Pt mixture before inspection using SEM. AFM images of imprints of H37Rv (E) and ΔlprG (F) in PDMS polymer. Pre-fixed bacilli were dried on polystyrene glass slides and used as template stamps to press into pre-cured PDMS polymer. The polymer was cured, the stamp was peeled off, and then the geometrical shape of the imprints was extracted using AFM. (G) Dimensions of H37Rv and ΔlprG obtained with SEM and AFM. Each value is an average of nine bacilli ±SD. (TIF) [file ppat.1004376.s003.tif]

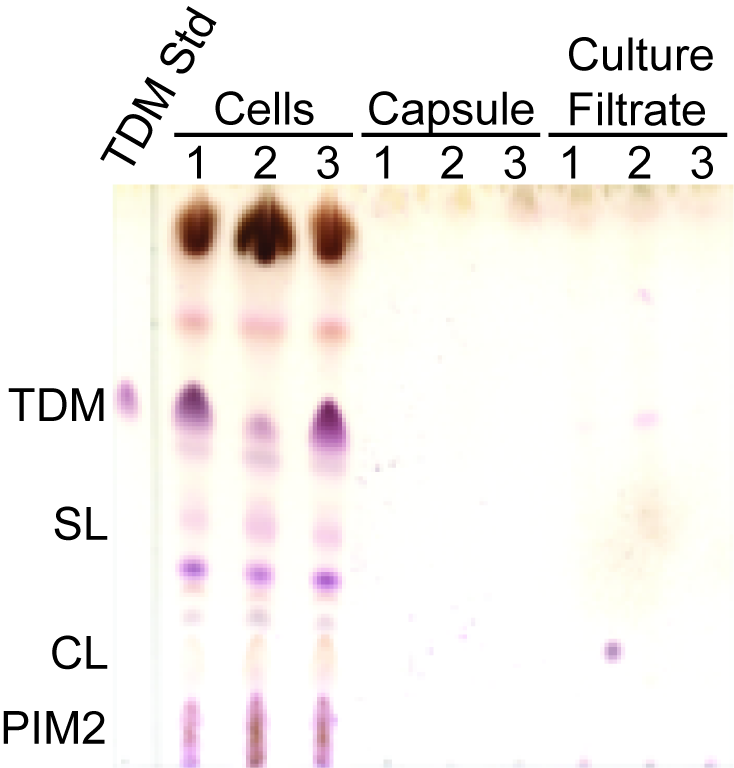

Supplement: Figure S4 — Phosphatidylinositol mannoside and trehalose dimycolate content of the lprG mutant. Thin-layer chromatograms of total lipids extracted from wild-type (H37Rv; 1), lprG mutant (ΔlprG; 2), and ΔlprG complemented with lprG-Rv1410c (::lprG; 3). The same amounts of total lipids were loaded for each strain. TLC plates were run in the solvent system CHCl3/CH3OH/H2O (20∶4∶0.5, by vol.) and revealed with α-naphthol. TDM, trehalose dimycolates; SL, sulfolipid; CL, cardiolipin; PIM2, phosphatidylinositol dimannosides; TDM Std, TDM standard. (TIF) [file ppat.1004376.s004.tif]

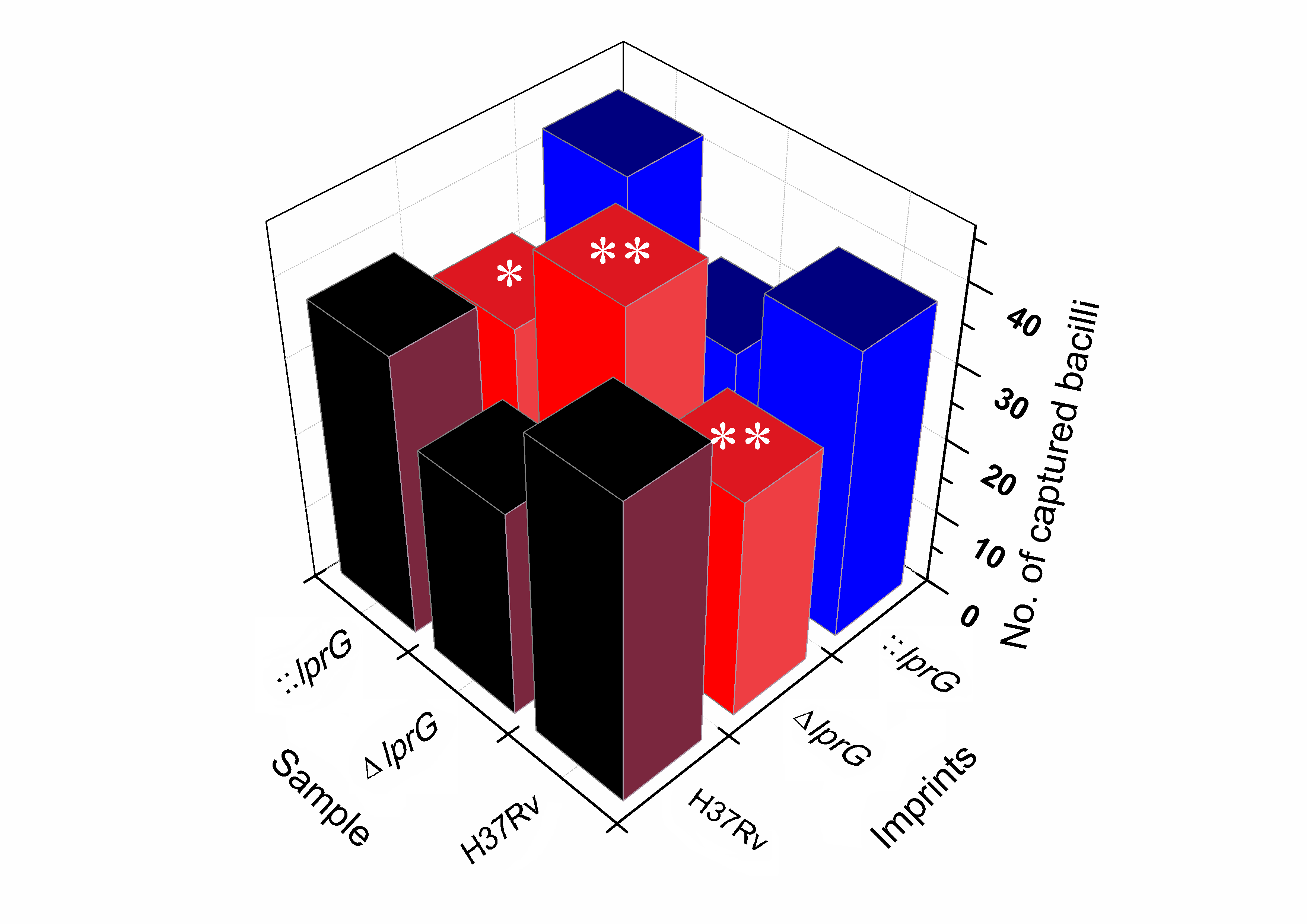

Supplement: Figure S5 — Cell-imprinting assay using bacteria pretreated with an isotype control. (A) Samples of wild-type (H37Rv), lprG mutant (ΔlprG), and ΔlprG complemented with lprG-Rv1410c (::lprG) suspended in PBS at an ODA580 of 0.01 were pretreated with rabbit IgG isotype control at a dilution of 1/10 and then fluorescently labeled with propidium iodide and 25 µl was flowed at 5 µl/min through a microfluidic device containing the imprints of H37Rv, ΔlprG, and ::lprG. The average number of captured bacilli per eight view fields was measured using a fluorescent microscope. Data is representative of three independent experiments. Capture on imprints of ΔlprG and ::lprG was compared to H37Rv. *P<0.05; **P<0.01. (TIF) [file ppat.1004376.s005.tif]
