# Supplementary material for: LprG-Mediated Surface Expression of Lipoarabinomannan Is Essential for Virulence of Mycobacterium tuberculosis
Source: PLoS Pathog. 2014 Sep 18;10(9):e1004376. doi: 10.1371/journal.ppat.1004376 (PMC4169494; doi:10.1371/journal.ppat.1004376)
Supplement: Table S1 — Number of H37Rv, Δ lprG , and :: lprG captured on cell-imprints of H37Rv, Δ lprG , and :: lprG . (DOC) [file ppat.1004376.s006.doc]

Table S1. Number of H37Rv, Δ*lprG*, and ::*lprG* captured on cell-imprints of H37Rv, Δ*lprG*, and ::*lprG*.

Average number of cells captured ±SD (P value vs. H37Rv)

Imprints

H37Rv Δ*lprG* ::*lprG* Δ*lspA* Δ*whiB3*

H37Rv 44.7 ± 4.0 34.0 ± 3.6 42.0 ± 4.0 39.3 ± 3.5 38.0 ± 3.6

Δ*lprG* 29.3 ± 3.5 (0.008) 58.3 ± 4.0 (0.002) 28.7 ± 4.0 (0.02) 30.3 ± 3.5 (0.04) 31.7 ± 3.5 (0.10)

::*lprG* 43.7 ± 4.2 (0.78) 35.7 ± 3.5 (0.60) 43.3 ± 3.5 (0.69) 39.7 ± 3.1 (0.91) 38.7 ± 3.5 (0.83)
