# Supplementary material for: LprG-Mediated Surface Expression of Lipoarabinomannan Is Essential for Virulence of Mycobacterium tuberculosis
Source: PLoS Pathog. 2014 Sep 18;10(9):e1004376. doi: 10.1371/journal.ppat.1004376 (PMC4169494; doi:10.1371/journal.ppat.1004376)
Supplement: Table S2 — Number of H37Rv, Δ lprG , and :: lprG captured on cell-imprints of H37Rv, Δ lprG , and :: lprG after pre-incubation of samples with anti-LAM polyclonal antibody α-LAM. (DOC) [file ppat.1004376.s007.doc]

Table S2. Number of H37Rv, Δ*lprG*, and ::*lprG* captured on cell-imprints of H37Rv, Δ*lprG*,

and ::*lprG* after pre-incubation of samples with anti-LAM polyclonal antibody α-LAM.

Average number of cells captured ± SD (P value vs. H37Rv)

Imprints

H37Rv Δ*lprG* ::*lprG* Δ*lspA* Δ*whiB3*

H37Rv 30.0 ± 3.6 32.3 ± 4.0 28.3 ± 3.5 27.7 ± 3.8 28.3 ± 3.1

Δ*lprG* 27.3 ± 3.5 (0.41) 54.7 ± 4.5 (0.003) 25.3 ± 3.5 (0.35) 27.0 ± 3.6 (0.84) 26.0 ± 3.6 (0.44)

::*lprG* 27.7 ± 3.1 (0.44) 33.7 ± 3.5 (0.69) 26.3 ± 3.5 (0.52) 25.7 ± 3.8 (0.55) 28.0 ± 3.0 (0.90)
