# Supplementary material for: LprG-Mediated Surface Expression of Lipoarabinomannan Is Essential for Virulence of Mycobacterium tuberculosis
Source: PLoS Pathog. 2014 Sep 18;10(9):e1004376. doi: 10.1371/journal.ppat.1004376 (PMC4169494; doi:10.1371/journal.ppat.1004376)
Supplement: Table S3 — Number of H37Rv, Δ lprG , and :: lprG captured on cell-imprints of H37Rv, Δ lprG , and :: lprG after pre-incubation of samples with anti-Ag85b complex monoclonal antibody CS-90. (DOC) [file ppat.1004376.s008.doc]

Table S3. Number of H37Rv, Δ*lprG*, and ::*lprG* captured on cell-imprints of H37Rv, Δ*lprG*,

and ::*lprG* after pre-incubation of samples with anti-Ag85b complex monoclonal antibody CS-90.

Average number of cells captured ±SD (P value vs. H37Rv)

Imprints

H37Rv Δ*lprG* ::*lprG*

H37Rv 42.0 ± 4.0 33.7 ± 4.2 38.7 ± 3.5

Δ*lprG* 28.7 ± 4.2 (0.02) 54.3 ± 4.5 (0.004) 26.3 ± 3.8 (0.01)

::*lprG* 39.3 ± 3.8 (0.45) 32.3 ± 3.5 (0.69) 39.0 ± 3.6 (0.91)
