# Supplementary material for: LprG-Mediated Surface Expression of Lipoarabinomannan Is Essential for Virulence of Mycobacterium tuberculosis
Source: PLoS Pathog. 2014 Sep 18;10(9):e1004376. doi: 10.1371/journal.ppat.1004376 (PMC4169494; doi:10.1371/journal.ppat.1004376)
Supplement: Table S4 — Number of H37Rv, Δ lprG , and :: lprG captured on cell-imprints of H37Rv, Δ lprG , and :: lprG after pre-incubation of samples with anti-LAM isotype control antibody. (DOC) [file ppat.1004376.s009.doc]

Table S4. Number of H37Rv, Δ*lprG*, and ::*lprG* captured on cell-imprints of H37Rv, Δ*lprG*,

and ::*lprG* after pre-incubation of samples with anti-LAM isotype control antibody.

Average number of cells captured ±SD (P value vs. H37Rv)

Imprints

H37Rv Δ*lprG* ::*lprG*

H37Rv 39.3 ± 3.1 29.0 ± 3.0 38.0 ± 4.4

Δ*lprG* 27.3 ± 3.1 (0.009) 43.0 ± 3.6 (0.007) 28.7 ± 3.1 (0.04)

::*lprG* 37.0 ± 3.0 (0.4) 31.7 ± 3.2 (0.35) 42.3 ± 4.2 (0.28)
