# Supplementary material for: LprG-Mediated Surface Expression of Lipoarabinomannan Is Essential for Virulence of Mycobacterium tuberculosis
Source: PLoS Pathog. 2014 Sep 18;10(9):e1004376. doi: 10.1371/journal.ppat.1004376 (PMC4169494; doi:10.1371/journal.ppat.1004376)
Supplement: Table S5 — Primers used in this study. (PDF) [file ppat.1004376.s010.pdf]

Table S5. Primers used in this study.

Primer Name	Forward Primer	Reverse Primer
Allelic Exchange Substrate		
lprG		
5′ AES	5′-cttaaggtgtgccgatccacaatggg-3′	5′-tctagaacagtggcggcgatgctaac-3′
3′ AES	5′-aagcttgggcgagaaggtccaggtca-3′	5′-actagtatcagccgggtgcgagcgaa-3′
Complementation		
lprG-Rv1410c operon-5′	5′-tctagaggttggaccggttcagtgt-3′	5′-ccatggcgatcgcggtcaac-3′
lprG-Rv1410c operon-3′	5′-ccatggtaatgatccacttc-3′	5′-aagcttaaggtcggaccggatctgtt-3′
RT-PCR		
lprG		
RT		5′-ctatctggttcaccgcctgt-3′
PCR	5′-cctgtacgccaccctgac-3′	5′-gttctggccgttgatggtat-3′
Rv1410c		
RT		5′-acaatccgaggatgttgtgc-3′
PCR	5′-tggttgatgtcgagctgttc-3′	5′-agtaaccgtacgccgcaata-3′

Restriction endonuclease sites are underlined
RT, reverse transcriptase
