# Supplementary material for: Correction: LprG-Mediated Surface Expression of Lipoarabinomannan Is Essential for Virulence of Mycobacterium tuberculosis
Source: PLoS Pathog. 2014 Oct 6;10(10):e1004489. doi: 10.1371/journal.ppat.1004489 (PMC4186873; doi:10.1371/journal.ppat.1004489)
Supplement: File S1 — Republished corrected article. (PDF) [file ppat.1004489.s001.pdf]

LprG-Mediated Surface Expression of Lipoarabinomannan Is Essential for Virulence of *Mycobacterium tuberculosis*

Rajiv L. Gaur[1]◑, Kangning Ren[2]◑, Antje Blumenthal[3], Suresh Bhamidi[4], Sara Gibbs[4], Mary Jackson[4,5,6,7], Richard N. Zare[2], Sabine Ehrt[3], Joel D. Ernst[8,9,10], Niaz Banaei[1,11]*

1 Department of Pathology, Stanford University, Stanford, California, United States of America, **2** Department of Chemistry, Stanford University, Stanford, California, United States of America, **3** Department of Microbiology and Immunology, Weill Cornell Medical College, New York City, New York, United States of America, **4** Mycobacteria Research Laboratories, Colorado State University, Fort Collins, Colorado, United States of America, **5** Department of Microbiology, Colorado State University, Fort Collins, Colorado, United States of America, **6** Department of Immunology, Colorado State University, Fort Collins, Colorado, United States of America, **7** Department of Pathology, Colorado State University, Fort Collins, Colorado, United States of America, **8** Department of Pathology, New York University School of Medicine, New York City, New York, United States of America, **9** Division of Infectious Diseases, Department of Medicine, New York University School of Medicine, New York City, New York, United States of America, **10** Department of Microbiology, New York University School of Medicine, New York City, New York, United States of America, **11** Division of Infectious Diseases and Geographic Medicine, Department of Medicine, Stanford University, Stanford, California, United States of America

Abstract

Mycobacterium tuberculosis employs various virulence strategies to subvert host immune responses in order to persist and cause disease. Interaction of *M. tuberculosis* with mannose receptor on macrophages via surface-exposed lipoarabinomannan (LAM) is believed to be critical for cell entry, inhibition of phagosome-lysosome fusion, and intracellular survival, but in vivo evidence is lacking. LprG, a cell envelope lipoprotein that is essential for virulence of *M. tuberculosis*, has been shown to bind to the acyl groups of lipoglycans but the role of LprG in LAM biosynthesis and localization remains unknown. Using an *M. tuberculosis lprG* mutant, we show that LprG is essential for normal surface expression of LAM and virulence of *M. tuberculosis* attributed to LAM. The *lprG* mutant had a normal quantity of LAM in the cell envelope, but its surface was altered and showed reduced expression of surface-exposed LAM. Functionally, the *lprG* mutant was defective for macrophage entry and inhibition of phagosome-lysosome fusion, was attenuated in macrophages, and was killed in the mouse lung with the onset of adaptive immunity. This study identifies the role of LprG in surface-exposed LAM expression and provides in vivo evidence for the essential role surface LAM plays in *M. tuberculosis* virulence. Findings have translational implications for therapy and vaccine development.

Citation: Gaur RL, Ren K, Blumenthal A, Bhamidi S, Gibbs S, et al. (2014) LprG-Mediated Surface Expression of Lipoarabinomannan Is Essential for Virulence of *Mycobacterium tuberculosis*. PLoS Pathog 10(9): e1004376. doi:10.1371/journal.ppat.1004376

Editor: Marcel A. Behr, McGill University, Canada

Received March 22, 2014; **Accepted** July 28, 2014; **Published** September 18, 2014

Copyright: © 2014 Gaur et al. This is an open-access article distributed under the terms of the Creative Commons Attribution License, which permits unrestricted use, distribution, and reproduction in any medium, provided the original author and source are credited.

Data Availability: The authors confirm that all data underlying the findings are fully available without restriction. All relevant data are within the paper and its Supporting Information files.

Funding: This work was supported by Stanford University Department of Pathology (NB) and NIH/NIAID AI064798 (MJ). The funders had no role in study design, data collection and analysis, decision to publish, or preparation of the manuscript.

Competing Interests: The authors declare that no competing interests exist.

* Email: niazbanaei@stanford.edu

◑ These authors contributed equally to this work.

Introduction

Mycobacterium tuberculosis is a human pathogen that has infected one-third of world's population and causes 8 million new cases and over one million deaths each year [1]. In the absence of a protective vaccine and the global prevalence of drug resistant strains, a greater understanding of virulence mechanisms is needed to facilitate development of anti-tuberculosis drugs with novel mechanisms of action. *M. tuberculosis* utilizes multiple defensive and offensive strategies to subvert the host immune responses in order to persist and cause disease [2]. Many of the virulence determinants of *M. tuberculosis* are constituents of the cell envelope [3]. The mycobacterial cell envelope is an impermeable barrier composed of an inner plasma membrane; a cell wall core; an outer mycomembrane; and a surface capsule composed of polysaccharides, lipids, and proteins [4–6]. The constituents of the capsule can be concealed within the ~35 nm capsular layer or they can be exposed on the surface with the ability to interact with the host [6]. Mannose-capped lipoarabinomannan (LAM), an abundant surface-exposed lipoglycan anchored to the inner and outer membranes via a mannosyl phosphate inositol [7], is considered one of the key virulence determinants of

Author Summary

Mycobacterium tuberculosis is among the leading infectious causes of human death. A better understanding of its virulence mechanisms is needed to facilitate development of novel therapeutics and a preventative vaccine. Lipoarabinomannan (LAM), an abundant surface-exposed lipoglycan, is believed to be a critical virulence determinant for intracellular survival and latency of *M. tuberculosis*. In vitro experiments with purified LAM have led to a model in which surface-exposed LAM binds to macrophage mannose receptor and facilitates bacterium entry, inhibition of phagosome-lysosome fusion, and modulation of innate immune responses. However, confirmation of these findings in vivo has not been possible due to the essentiality of genes involved in the LAM biosynthetic pathway. It was recently shown that LprG, a cell envelope lipoprotein, binds to the acyl groups of lipoglycan, but the role of LprG in LAM biosynthesis and localization remains unknown. Here, using an *M. tuberculosis lprG* mutant and a novel cell-imprinting assay, we show that LprG is essential for normal surface expression of LAM and virulence of *M. tuberculosis* attributed to LAM. Our study provides new insights into the mechanism of surface expression of LAM and confirms the essential role surface LAM serves in pathogenesis of *M. tuberculosis*.

M. tuberculosis [6,8–11]. Upon contact with macrophages, surface-exposed LAM binds to macrophage mannose receptor (MMR) and facilitates entry, inhibition of Phagosome-Lysosome (P-L) fusion, and modulation of immune responses leading to intracellular survival and persistence [12–16]. However, our understanding of LAM function is derived from in vitro studies using purified LAM. Confirmation of these findings in vivo using *M. tuberculosis* mutants has not been possible due to the essentiality of genes involved in lipoglycan biosynthetic pathway [8,9].

Bacterial lipoproteins are membrane-anchored cell envelope proteins with a broad range of functions including substrate-binding and transport [17]. Using a Toll-like receptor 2 assay to detect agonist activity from mycobacterial lysates, Drage and colleagues unexpectedly found that *M. tuberculosis* lipoprotein LprG binds to LAM and other lipoglycans [18]. Using X-ray crystallography, they showed that LprG forms a hydrophobic pocket that accommodates the alkyl chains of tri-acylated lipoglycans and mutation of the hydrophobic pocket (LprG V91W) abrogates association of LprG with lipoglycans. The genome of *M. tuberculosis* encodes *lprG* (Rv1411c) in an operon that also encodes Rv1410c, a major facilitator superfamily small molecule transporter P55 [19]. Both LprG and P55 are required for the efflux activity of P55 using exogenous substrates suggesting that both proteins mediate the same endogenous function [20,21]. Although *lprG* is essential for survival of *M. tuberculosis* in mice [22,23], the function of LprG in LAM biosynthesis and localization remains unknown.

In this study we used a *M. tuberculosis lprG* deletion mutant to investigate the role of LprG in LAM biosynthesis and pathogenesis. We show that (i) LprG is essential for normal expression of surface LAM and (ii) the virulence properties of *M. tuberculosis* attributed to LAM are defective in the *lprG* mutant.

Results

Fitness of *lprG* mutant is unaltered in broth culture

To investigate the function of LprG in *M. tuberculosis*, we constructed a deletion mutant of *lprG* (Δ*lprG*) in H37Rv and complemented it with *lprG*-Rv1410c (::*lprG*) (Figure S1). Deletion of *lprG* had no effect on the growth of *M. tuberculosis* in Middlebrook 7H9 broth (Figure S2A). On Middlebrook 7H9 agar, the diameter of Δ*lprG* colonies was on average reduced by 47% compared to H37Rv and ::*lprG* (Figure S2B). To investigate the length and diameter of Δ*lprG* bacilli, scanning electron microscopy (SEM) and atomic force microscopy (AFM) were performed on the bacteria and their imprints on polydimethylsiloxane (PDMS) polymer surface, respectively. SEM and AFM did not show any dimension differences between H37Rv and Δ*lprG* (Figure S3). Both strains measured ~2.4 μm long and 0.27 μm wide by SEM and had an imprinted depth of 0.26 μm.

lprG mutant has normal LAM content

To investigate the role of LprG in LAM biosynthesis and transport, the cellular, capsular, and secreted lipoglycan and lipid composition of Δ*lprG* were analyzed in non-shaking and shaking cultures without detergent. As shown in Figure 1A and B, using SDS/PAGE stained with periodic acid/Schiff reagent and thin-layer chromatography, comparable amounts of LAM and its precursors- phosphatidylinositol mannosides and lipomannan- were recovered from H37Rv and Δ*lprG* cells. The phosphatidylinositol mannosides composition as determined by MALDI-TOF MS was identical (unpublished data). As a complementary approach, we assessed the cellular LAM content using SDS/PAGE immunoblot. The immunoblot showed no difference between H37Rv and Δ*lprG* (Figure 1C). Consistent with these observations, the mannose, inositol, and arabinose composition of whole delipidated Δ*lprG* cells was not significantly different from that of the H37Rv (Table 1). Likewise, both strains displayed similar glycan contents in their culture filtrate and capsular materials indicating that their capsular lipoglycan compositions were not altered [24]. Biochemical methods also did not reveal any significant quantitative or qualitative differences in the lipid composition between H37Rv and Δ*lprG*, apart from a decrease in trehalose dimycolate in the *lprG* mutant cells growing in shaking broth but not on agar (Figure 1B and Figure S4). We also assessed the major capsular polysaccharide α-glucan by a spot immunoblot assay and did not find a difference between H37Rv and Δ*lprG* (Figure 1D). Altogether, these findings consistently indicate that LprG does not play a role in the biogenesis of LAM.

lprG mutant has reduced expression of surface-exposed LAM

To investigate the surface property of Δ*lprG* and determine the role of LprG in expression of LAM on the surface [10], we employed cell-imprinting technology [25,26]. As illustrated in Figure 2A, cell-imprinting cures PDMS polymer solution around bacteria of interest. During contact, functional groups in the polymer solution arrange around the template cells. After removing the template cells, the cavities left on the imprinting polymer surface serve as receptors for preferential capture of template cells based on the chemical property of their surface. When cells of Δ*lprG* were passed through a microfluidic device containing imprints of H37Rv, Δ*lprG*, and ::*lprG*, the mutant cells were selectively captured on imprints of Δ*lprG* compared to imprints of H37Rv and ::*lprG* (Figure 2B and C). On average 58.3±4.0 Δ*lprG* cells were captured per field on imprint of Δ*lprG* compared to 34.0±3.6 ($P<0.005$) and 35.7±3.5 ($P<0.005$) Δ*lprG* cells captured on imprints of H37Rv and ::*lprG*, respectively (Table S1). In contrast, the cells of H37Rv and ::*lprG* were preferentially captured on imprints of H37Rv and ::*lprG* compared to imprints of Δ*lprG* (Figure 2B and C). On average 44.7±4.0 ($P<0.01$) and 43.7±4.2 ($P<0.05$) H37Rv cells and

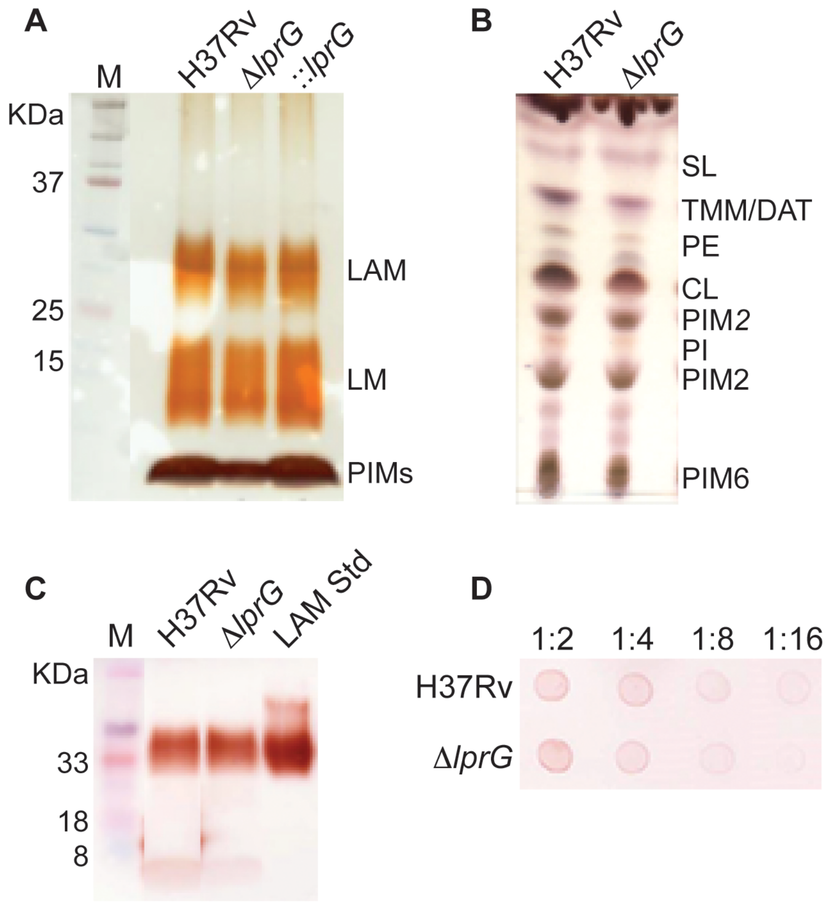

Figure 1. The *lprG* mutant has normal LAM content in the cell envelope. (A) SDS/PAGE analysis of phosphatidylinositol mannosides (PIMs), lipomannan (LM) and LAM prepared from wild-type (H37Rv), *lprG* mutant (Δ*lprG*), and Δ*lprG* complemented with *lprG*-Rv1410c (::*lprG*). LM and LAM extracted from equal amounts of bacterial cells were separated on a 10–20% Tricine gel and visualized by periodic acid/Schiff reagent staining. (B) Thin-layer chromatograms of total lipids extracted from H37Rv and Δ*lprG*. The same amounts of total lipids extract from bacilli grown in GAS medium were loaded for each strain. Thin-layer chromatogram plates were run in the solvent system $CHCl_3/CH_3OH/H_2O$ (65:25:4, by vol.) and revealed with α-naphthol. SL, sulfolipid; TMM, trehalose monomycolates; DAT, diacyltrehaloses; PE, phosphatidylethanolamine; CL, cardiolipin; PIM_2, phosphatidylinositol dimannoside; PI, phosphatidylinositol; PIM_6, phosphatidylinositol hexamannosides. (C) SDS/PAGE immunoblot for LAM analysis in H37Rv and Δ*lprG* cellular extracts. Extracts normalized to protein concentration were separated on a 15% SDS/PAGE gel and transferred to PVDF membrane. The blot was blocked, and then stained with anti-LAM pAb (α-LAM) followed by goat anti-rabbit IgG-HRP secondary antibody. The blot was washed and imaged after adding 30% 3,3′-diaminobenzidine tetrahydrochloride solution plus 0.0005% H_2O_2. LAM Std, purified H37Rv LAM standard. (D) Spot immunoblot for analysis of capsular α-glucan. Capsular content extracted from equal numbers of bacteria were spotted on PVDF membrane and stained with goat anti-phosphatidylinositol-glycans pAb followed by donkey anti-goat IgG-HRP secondary antibody. The membrane was developed and imaged as described in C. Dilutions of extract spotted on membrane are shown. Data is representative of two independent experiments.
doi:10.1371/journal.ppat.1004376.g001

42.0±4.0 (P<0.05) and 43.3±3.5 (P<0.01) ::*lprG* cells were captured on imprints of H37Rv and ::*lprG*, respectively, compared to 29.3±3.5 H37Rv and 28.7±4.0 ::*lprG* cells captured on imprints of Δ*lprG* (Table S1). These results confirm that Δ*lprG* has an altered surface compared to H37Rv and ::*lprG*. To determine whether the cell surface alteration in Δ*lprG* is a common feature of other cell envelope mutants, we measured the capture of *M. tuberculosis* Δ*lspA* [27] and Δ*whiB3* [28] on imprints of H37Rv, Δ*lprG*, and ::*lprG*. As shown in Figure 2C, both Δ*lspA* and Δ*whiB3* cells were captured preferentially on imprints of H37Rv and ::*lprG* compared to imprints of Δ*lprG*. On average 39.3±3.5, 30.3±3.5 (P<0.05), and 39.7±3.1 Δ*lspA* cells and 38.0±3.6, 31.7±3.5, and 38.7±3.5 Δ*whiB3* cells were captured on imprints of H37Rv, Δ*lprG*, and ::*lprG*, respectively (Table S1). These findings indicate the cell surface alteration in Δ*lprG* is not a common feature of cell envelope mutants.

To determine if the altered surface property of Δ*lprG* is caused by differential expression of surface LAM, bacteria were pretreated with anti-LAM antibody and then captured on imprints. Blocking surface LAM with two different anti-LAM antibodies, α-LAM and CS-35, abolished selective capture of H37Rv and ::*lprG* on imprints of H37Rv and ::*lprG* compared to imprint of Δ*lprG* (Figure 2D and unpublished data). On average 30.0±3.6, 27.3±3.5, and 27.7±3.1 H37Rv cells and 28.3±3.5, 25.3±3.5, and 26.3±3.5 ::*lprG* cells were captured on imprints of H37Rv, Δ*lprG*, and ::*lprG*, respectively, after blocking with α-LAM (Table S2). Pretreatment of Δ*lprG* cells with anti-LAM had no effect on selective capture of Δ*lprG* cells on imprint of Δ*lprG* compared to imprints of H37Rv and ::*lprG*. On average 32.3±4.0, 54.7±4.5 (P<0.005), and 33.7±3.5 Δ*lprG* cells were captured on imprints of H37Rv, Δ*lprG*, and ::*lprG*, respectively (Table S2). To show that the blocking effect of anti-LAM was specific, bacteria were pretreated with an antibody to Ag85c complex, a cell

Table 1. The glycosyl composition of the delipidated cells, capsules and culture filtrates of the H37Rv and *lprG* mutant (Δ*lprG*) strains.

Source	Strain	Ino+Ara+Man/total sugars	Glc/total sugars	Ino/total sugars	Ara/total sugars	Man/total sugars
Delipidated cells						
	H37Rv	0.23	0.34	0.02	0.07	0.14
	Δ*lprG*	0.28	0.32	0.03	0.10	0.15
Capsule						
	H37Rv	0.17	0.82	0.02	0.02	0.14
	Δ*lprG*	0.14	0.84	0.02	0.02	0.10
Culture filtrates						
	H37Rv	0.47	0.30	0.14	0.13	0.20
	Δ*lprG*	0.48	0.28	0.15	0.14	0.18

Ino, *myo*-Inositol; Ara, arabinose; Man, mannose; Glc, glucose.
Values are expressed as molar ratios.
doi:10.1371/journal.ppat.1004376.t001

wall antigen not expressed on the surface. Treatment of bacteria with anti-Ag85c complex had no effect on selective capture of H37Rv or ::*lprG* on H37Rv and ::*lprG* imprints (Figure 2E and Table S3). Similar results were obtained with an isotype control for CS-35 (Figure S5 and Table S4).

To generate additional evidence for altered expression of surface LAM on Δ*lprG*, we used indirect fluorescent antibody staining to measure the expression of surface-exposed LAM. Surface staining with anti-LAM, but not secondary antibody alone, showed a lower percent of Δ*lprG* cells staining positive with α-LAM and CS-35 compared to H37Rv and ::*lprG* (Figure 3). On average 45.3±0.18% and 41.3±4.2% of Δ*lprG* cells stained positive with α-LAM and CS-35, respectively, compared to 81.4±3.6% ($P<0.001$) and 86.0±14.3% ($P<0.01$) of H37Rv cells and 84.1±6.0% ($P<0.01$) and 73.1±17.8% ($P<0.05$) of ::*lprG* cells, respectively. A higher fraction of positively staining Δ*lprG* cells stained dim compared to H37Rv and ::*lprG*. On average 92.0% and 100% of Δ*lprG* stained dim with α-LAM and CS-35, respectively, compared to 48.9% and 40.7% of H37Rv and 66.0% and 36.0% of ::lprG, respectively. Altogether, a notable difference between H37Rv and Δ*lprG* was the abundance of surface-exposed LAM, indicating LprG is important for normal surface expression of LAM.

lprG mutant is attenuated for macrophage entry and inhibition of Phagosome-Lysosome fusion

Interaction of *M. tuberculosis* with the host through binding of surface LAM to MMR on macrophages facilitates cell entry and inhibition of P-L fusion [14–16]. Thus, we measured the efficiency of Δ*lprG* for cell entry and inhibition of P-L fusion. To measure cell entry, we infected mouse bone marrow-derived macrophages (BMMΦ) and determined the numbers of intracellular viable bacteria 4 h after infection. As shown in Figure 4A, infection with similar numbers of organisms of each strain resulted in recovery of a significantly lower number of the Δ*lprG* compared to H37Rv and ::*lprG*. On average 1,114±277 Δ*lprG* cells were recovered per well compared to 3,363±992 ($P<0.05$) H37Rv and 2,256±431 ($P<0.05$) ::*lprG* cells. To show that the superior entry of H37Rv and ::*lprG* was dependent on their interaction with MMR, we used 4 mg/ml mannan and 0.5 μg/ml anti-MMR to block MMR on RAW 264.7 macrophages prior to infection. Without blocking, 18.2±8.4% of RAW cells were infected with Δ*lprG* compared to 59.7±5.4% ($P<0.05$) with H37Rv and 51.1±4.3% ($P<0.05$) with ::*lprG* (Figure 4B). Blocking the MMR with mannan (Figure 4C) and anti-MMR (Figure 4D) abolished differences in the percentage of RAW cells infected with H37Rv and Δ*lprG*. On average 5.7±1.0% and 3.8±1.4% of cells were infected with H37Rv and 6.5±2.2% and 6.3±1.5% of cells were infected with Δ*lprG* after treatment with mannan and anti- MMR, respectively.

To investigate P-L fusion, we measured the percentage of intracellular bacteria in RAW cells that co-localized with lysosomes stained with LysoTracker-Red. As shown in confocal images (Figure 5A) and in a graph (Figure 5B), co-localization of Δ*lprG* with lysosomes was increased compared to H37Rv and ::*lprG*. On average 71.3±3.3% of Δ*lprG* co-localized with lysosomes compared to 48.6±8.7% ($P<0.05$) of H37Rv and 44.7±5.8% ($P<0.01$) of ::*lprG*. Blocking the MMR with mannan (Figure 5C) and anti-MMR (Figure 5D) abolished co-localization differences between H37Rv and Δ*lprG*. On average 86.6±2.2% and 89.6±5.8% of H37Rv and 92.7±7.3% and 81.3±12.5% of Δ*lprG* co-localized with lysosomes in cells treated with mannan and anti-MMR, respectively. These findings confirm that LprG is essential for optimal macrophage entry and inhibition of P-L fusion.

lprG mutant burden declines in the mouse lung with the onset of adaptive immunity

To characterize the kinetics of Δ*lprG* replication in the mouse lung model, we infected wild-type C57BL/6 mice with aerosolized bacteria and assessed bacterial burden and lung pathology during acute and chronic phases of infection. As shown in Figure 6A, Δ*lprG* was attenuated during the acute and chronic phases of infection. Although Δ*lprG* could replicate in the mouse lung, on day 10 the burden of Δ*lprG* was on average 8.3 and 14.5 fold lower than H37Rv ($P<0.01$) and ::*lprG* ($P<0.01$), respectively. With the onset of adaptive immunity [29], the burden of Δ*lprG* started to decline. On day 70, the burden of Δ*lprG* was reduced by 2,805 and 2,721 fold compared to H37Rv ($P<0.005$) and ::*lprG* ($P<0.005$), respectively. Gross examination on day 70 revealed normal sized lungs with no or small lesions in mice infected with Δ*lprG* (Figure 6B). In contrast, mice infected with H37Rv and ::*lprG* had edematous lungs with numerous large granulomatous lesions.

Attenuation of *lprG* mutant is not rescued in p-47phox−/− and iNos−/− macrophages

To determine whether the mechanism of in vivo attenuation of Δ*lprG* is due to hypersensitivity to reactive oxygen or nitrogen species, we investigated the growth and survival of Δ*lprG* in BMMΦ

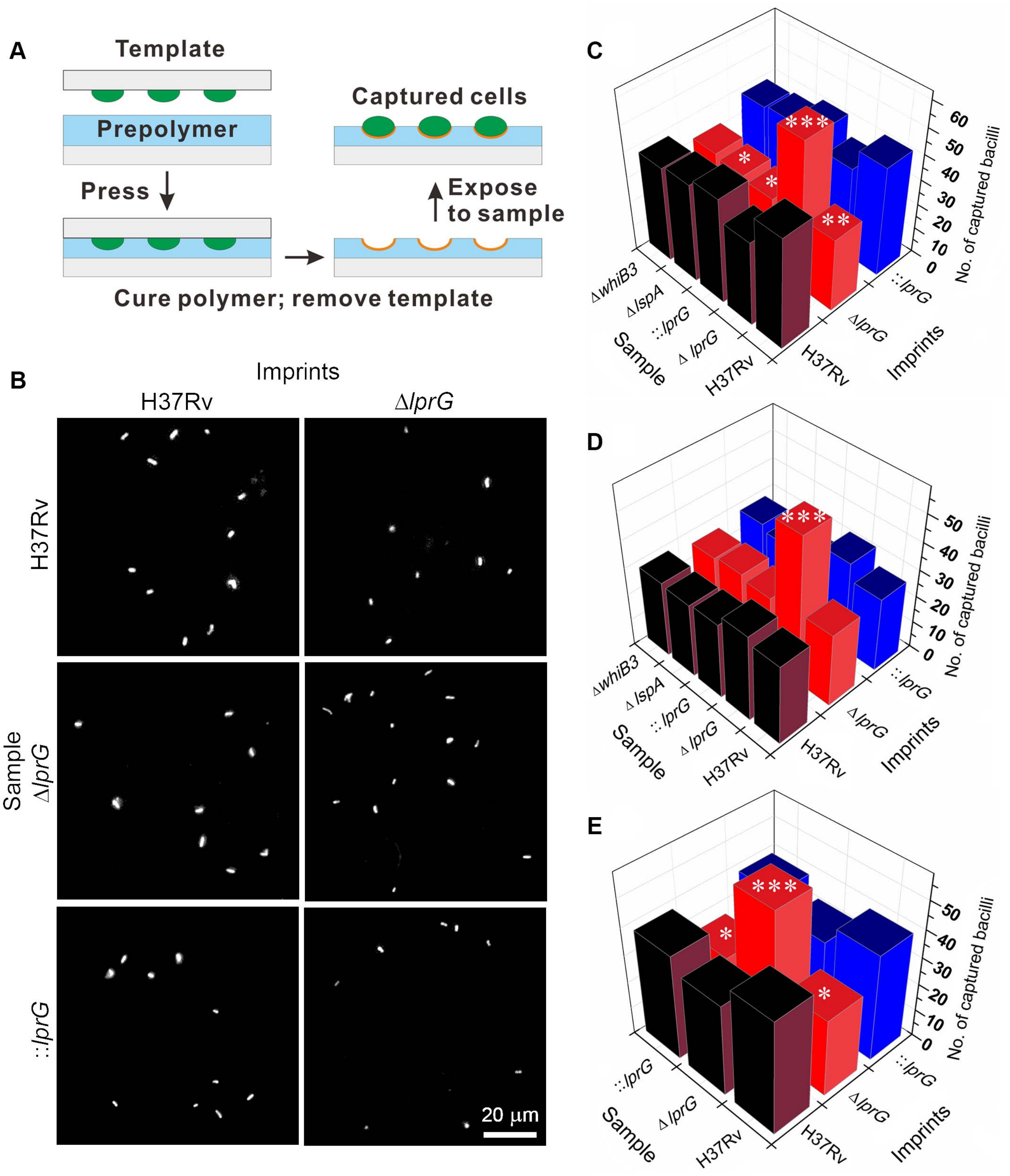

Figure 2. Cell-imprinting reveals altered cell surface and reduced surface-exposed LAM in the *lprG* mutant. (A) Illustration of cell-imprinting and cell capture. Equal number of bacteria grown in shaking broth culture with Tween-80 were fixed in paraformaldehyde, washed with PBS, dried on a polystyrene glass slide, and then used as a template for imprinting. The template was pressed into pre-cured PDMS polymer which was then cured at 37°C for 8 h, followed by 60°C for 1 h. The template was peeled off and the imprinted polymer film was sonicated in distilled water for 5 min. Samples of bacteria suspended in PBS at an OD_{A580} of 0.01 were fluorescently labeled with propidium iodide and 25 μl was flowed at 5 μl/min through a microfluidic device containing the imprints of wild-type (H37Rv), *lprG* mutant (Δ*lprG*), and Δ*lprG* complemented with *lprG*-Rv1410c (::*lprG*). The average number of captured bacilli per eight view fields was measured using a fluorescent microscope. (B) Representative images showing captured H37Rv, Δ*lprG*, and ::*lprG* on imprints of H37Rv and Δ*lprG*. (C–E) Bar graphs show the average number of captured H37Rv, Δ*lprG*, ::*lprG*, *lspA* mutant (Δ*lspA*) and *whiB3* mutant (Δ*whiB3*). Samples were pretreated with PBS alone (C), with rabbit anti-LAM pAb (α-LAM) at a dilution of 1/20 (D) and with mouse anti-Ag85 complex mAb (CS-90) at a dilution of 1/10 (E). Data is representative of three independent experiments. Capture on imprints of Δ*lprG* and ::*lprG* was compared to H37Rv. *P<0.05; **P<0.01; ***P<0.005.
doi:10.1371/journal.ppat.1004376.g002

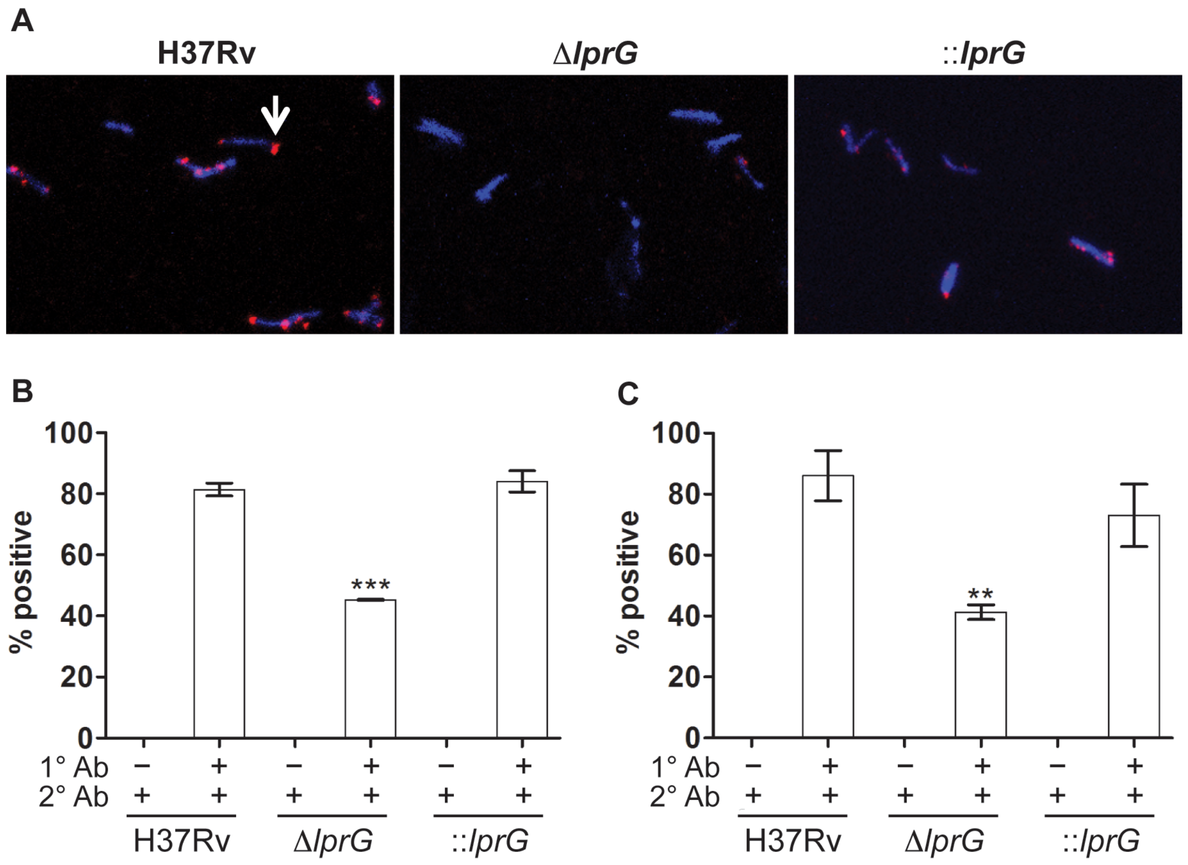

Figure 3. Surface staining reveals reduced LAM on the *lprG* mutant cells. Indirect fluorescent antibody staining of wild-type (H37Rv), *lprG* mutant (Δ*lprG*), and Δ*lprG* complemented with *lprG*-Rv1410c (::*lprG*) with anti-LAM antibodies. Bacilli cultured in shaking broth cultures were washed, fixed in 4% paraformaldehyde, and then suspended in 5% bovine serum albumin for 1 h. Bacilli were incubated with primary antibodies (1° Ab) rabbit anti-LAM pAb (α-LAM) and mouse anti-LAM mAb (CS-35), washed, and then incubated with secondary antibodies (2° Ab), goat anti-rabbit IgG Fab2-Alexa fluor and goat anti-mouse IgG IgG-Dylight, respectively. Bacilli were mounted with DAPI-containing medium and visualized using a fluorescent microscope. (A) Confocal images showing positive staining bacteria with α-LAM (arrow). (B and C) Bar graphs show average percent of bacteria staining positive with α-LAM (B) and CS-35 (C) ±SD of three independent experiments. Δ*lprG* and ::*lprG* were compared to H37Rv. **$P<0.01$; ***$P<0.001$.
doi:10.1371/journal.ppat.1004376.g003

from wild-type (C57BL/6), p-47phox−/−, and iNOS−/− mice. Intracellular bacilli in resting and IFN-γ-activated BMMΦ were enumerated on day 0, 2 and 5 post-infection. The *lprG* mutant was attenuated in resting and IFN-γ-activated wild-type macrophages (Figure 7A, B, E and F). Because Δ*lprG* is attenuated for cell entry, we determined the fold-change in colony forming units (CFU) between day 0 and 5. On average, fold-change in Δ*lprG* was 5.2 and 1.1 in resting and IFN-γ-activated macrophages, respectively, compared to 16.5 ($P<0.001$) and 3.2 ($P<0.01$) in H37Rv and 14.3 ($P=0.07$) and 3.5 ($P<0.05$) in ::*lprG*, respectively. As shown in Figure 7, attenuation of Δ*lprG* was not reversed in p-47phox−/− (Figure 7C and D) or iNOS−/− (Figure 7G and H) macrophages. On day 5, on average, 8,767 and 1,183 Δ*lprG* were recovered per well from resting and IFN-γ-activated p-47phox−/− macrophages, respectively, compared to 9,667 and 1,683 from wild-type macrophages, respectively. In iNOS−/− macrophages, 138,333 and 35,000 Δ*lprG* were recovered from resting and IFN-γ-activated macrophages, respectively, compared to 108,333 and 26,667 from wild-type macrophages, respectively.

Discussion

Here we provide evidence that LprG is essential for normal surface expression of LAM in *M. tuberculosis*. We showed that the *lprG* mutant has normal abundance of lipids, lipoglycans, and glycans in the cell wall and the capsule, but it has an altered surface with reduced expression of surface-exposed LAM. This finding is consistent with prior studies showing the *lprG* mutant of *M. smegmatis* is defective for colony sliding motility [21] and of *M. bovis* BCG displays increased clumping in broth [30], both of which are properties associated with cell surface alteration. The exact mechanism through which LprG and P55 facilitate normal surface expression of LAM remains to be determined. The finding that LAM does not accumulate in the cell envelope or culture filtrate of the *lprG* mutant rules out the possibility that LprG plays a role in transport or anchoring of LAM, respectively. Furthermore, normal migration of LAM on SDS/PAGE blots together with unchanged capsular glycan content suggests LprG is not involved in elaboration of the LAM side-chains. Drage and colleagues showed that LprG associates with LAM through a hydrophobic pocket that accommodates the alkyl chains of triacylated lipoglycans [18]. Thus, we speculate LprG serves to orient LAM on the cell surface in order to maximize exposure of its mannose cap residues. This would be consistent with the paradigm that *M. tuberculosis* depends on surface-exposed LAM to bind to MMR in order to facilitate cell entry and inhibition of P-L fusion [14–16]. Misorientation of surface LAM in the *lprG* mutant would also explain reduced but not null expression of

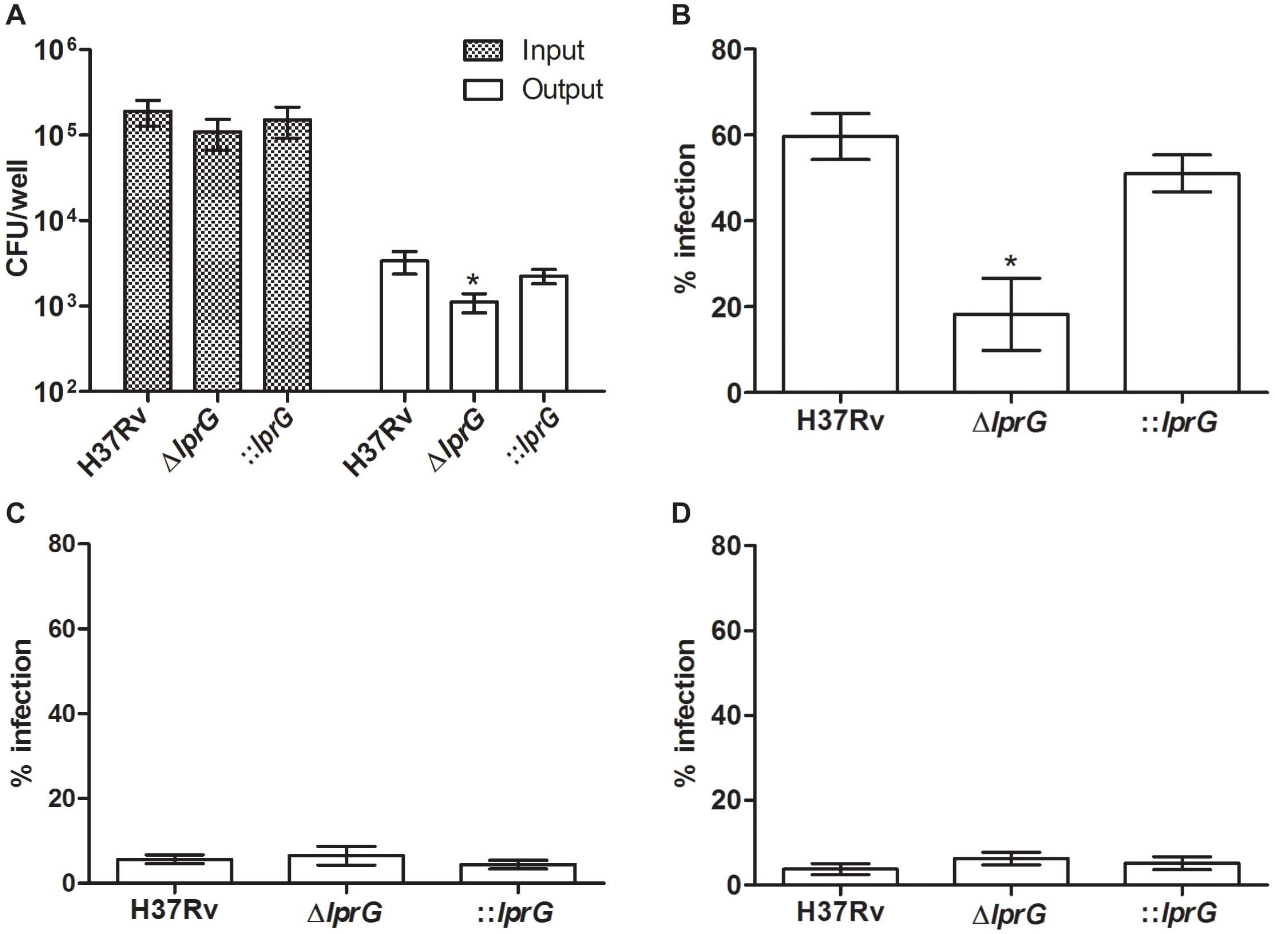

Figure 4. The *lprG* mutant is impaired for macrophages entry. (A) Resting BMMΦ from C57BL/6 mice were infected with wild-type (H37Rv), *lprG* mutant (Δ*lprG*), and Δ*lprG* complemented with *lprG*-Rv1410c (::*lprG*) at MOI of 1:10 (Input). At 4 h post-infection cells were washed and intracellular bacteria (Output) were enumerated on agar. Bar graph shows average CFU counts ±SEM of three independent experiments performed in triplicate wells. (B–D) RAW 264.7 macrophages treated with and without 4 mg/ml mannan or 0.5 μg/ml anti-MMR pAb were infected with FITC-labeled bacteria at MOI of 1:100 for 2 h. Cells were washed, mounted and visualized using a fluorescent microscope. Graphs show percentage of infected cells with no treatment (B), treated with mannan (C) and treated with anti-MMR pAb (D). Bars show average percentage ±SEM of two independent experiments performed in triplicates. Δ*lprG* and ::*lprG* were compared to H37Rv. *$P<0.05$.
doi:10.1371/journal.ppat.1004376.g004

surface LAM based on anti-LAM staining. Alternatively, LprG may be essential for minor modification of the LAM side-chains, which would impact normal surface chemistry and LAM immunostaining. We also showed that the *lprG* mutant has a smaller colony size on agar. The exact role of surface LAM in colony size remains to be determined but the phenotype may be due to the altered surface property of the *lprG* mutant. Alternatively, LprG could have a secondary function that is unrelated to orienting LAM.

Prior studies using purified mannose-capped LAM from *M. tuberculosis* have identified it as a key virulence determinant for cell entry and inhibition of P-L fusion, which presumably leads to intracellular survival and persistence [8]. However, until now, confirmation of these virulence properties in LAM mutants had not been possible due to the essentiality of genes in the LAM biosynthetic pathway [8,9]. Here we characterized the functional consequences of surface LAM deficiency in the *lprG* mutant. We showed that the *lprG* mutant is in fact less efficient at macrophage entry and inhibition of P-L fusion, and is attenuated for replication in macrophages. These findings are consistent with the attenuated phenotype of the *lprG* mutant reported in mice [22,23]. Our study extends the observations made in vivo by providing a more detailed characterization of the mutant in mice after aerosol infection, which more closely approximates natural infection in humans. We showed that while the *lprG* mutant is able to survive and partially replicate in the mouse lung during the acute phase of infection when innate immunity is in operation, the mutant is killed in the lung with the onset of adaptive immunity [29]. The attenuation during the acute phase may be ascribed to the deficiency of the mutant to enter host cells. We also showed that the attenuation of the *lprG* mutant in resting and IFN-γ-activated wild-type macrophages is not rescued in p47phox−/− or iNOS−/− macrophages, indicating that the *lprG* mutant is not hypersensitive to reactive oxygen and nitrogen species, respectively. This finding is inconsistent with the observation by Chan and colleagues that LAM has the property of scavenging oxygen free radicals [31]. Perhaps the orientation of LAM on the surface is not essential for the scavenging property. However, confirmation of our findings in knockout mice is essential because enhanced replication of H37Rv or ::*lprG* in either p47phox−/− or iNOS−/− macrophages was not observed. The *lprG* mutant also has a cell envelope defect based on increased susceptibility to

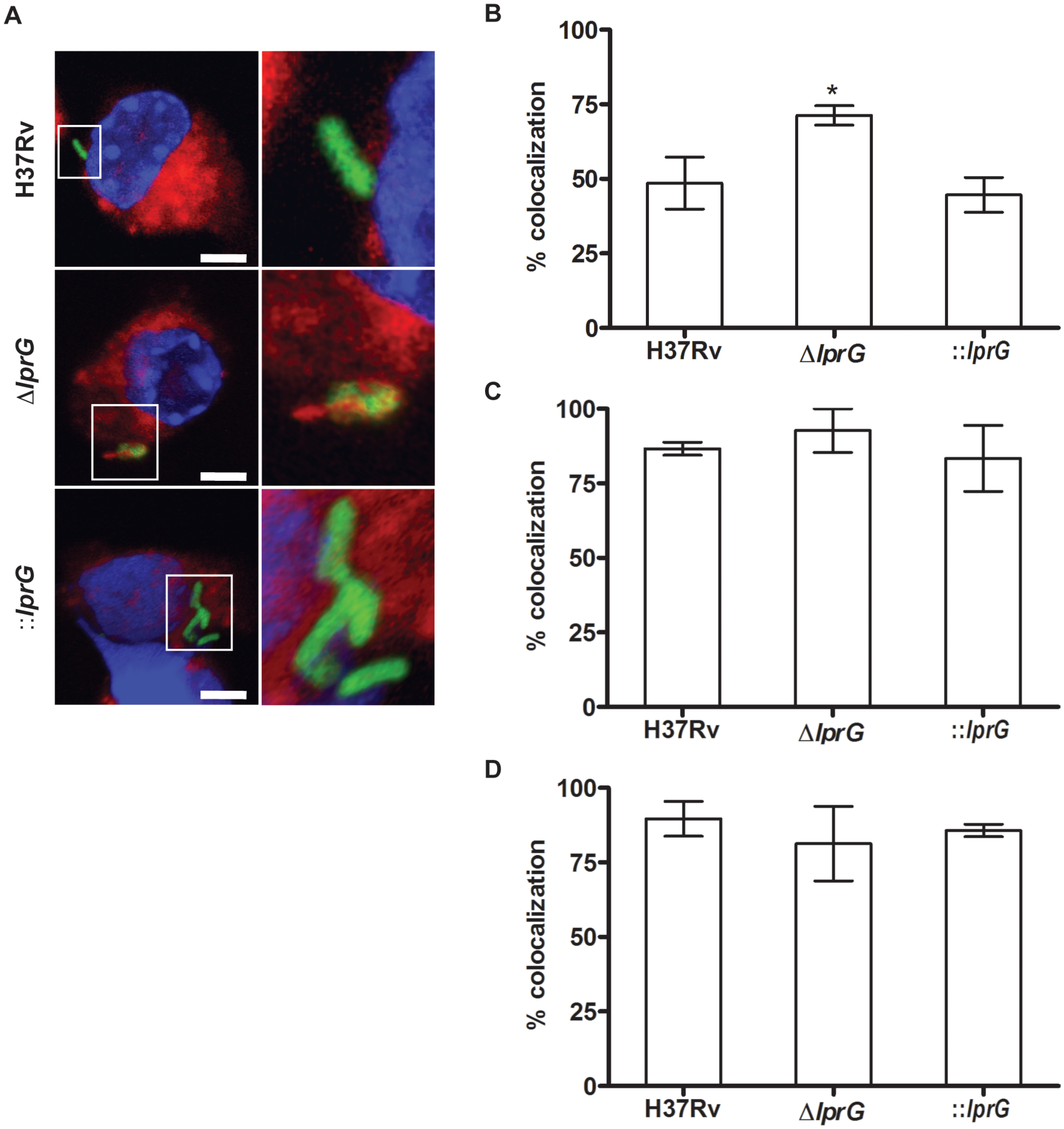

Figure 5. The *lprG* mutant is impaired for inhibition of Phagosome-Lysosome fusion. RAW 264.7 cells were treated with 50 nM LysoTracker-Red with and without 4 mg/ml mannan or 0.5 μg/ml anti-MMR pAb. Cells were incubated for 30 min, and then infected with FITC-labeled wild-type (H37Rv), *lprG* mutant (Δ*lprG*), and Δ*lprG* complemented with *lprG*-Rv1410c (::*lprG*) at MOI of 1:100 for 2 h. Cells were washed, mounted, and visualized using a fluorescent microscope. (A) Confocal images showing bacteria in green, lysosomes in red, and the nucleus in blue. Scale bars, 5 μm. 2.5× magnification is shown on the right. (B–D) Graphs show average percentage of bacilli co-localized with lysosomes in cells with no treatment (B), treated with mannan (C) and treated with anti-MMR pAb (D). Bars show average percentage ±SEM of two to three independent experiments performed in triplicates. Δ*lprG* and ::*lprG* were compared to H37Rv. *$P<0.05$.
doi:10.1371/journal.ppat.1004376.g005

SDS which suggests the in vivo attenuation of the *lprG* mutant may be, in part, due to permeability to immune effectors such as antimicrobial peptides [20]. Other potential mechanisms of attenuation not investigated in this study include loss of LAM-dependent inhibition of protein kinase C activity [31], expansion of regulatory T cells [32], and suppression of a dendritic cell pro-inflammatory program [33]. Furthermore, although our results strongly indicate LAM orientation is the major factor that modulates the surface chemical property of the *lprG* mutant, we are unable to rule out the possibility that interaction of LprG with phosphatidylinositol mannosides, lipomannan, and/or other lipoglycan contributes to the phenotypes observed in the *lprG* mutant.

The bacteria used for cell-imprinting and surface staining experiments in this study were grown under perturbing conditions (in the presence of 0.05% Tween-80 and mechanical agitation). Using cryo-electron microscopy (EM) and immuno gold-EM, Sani

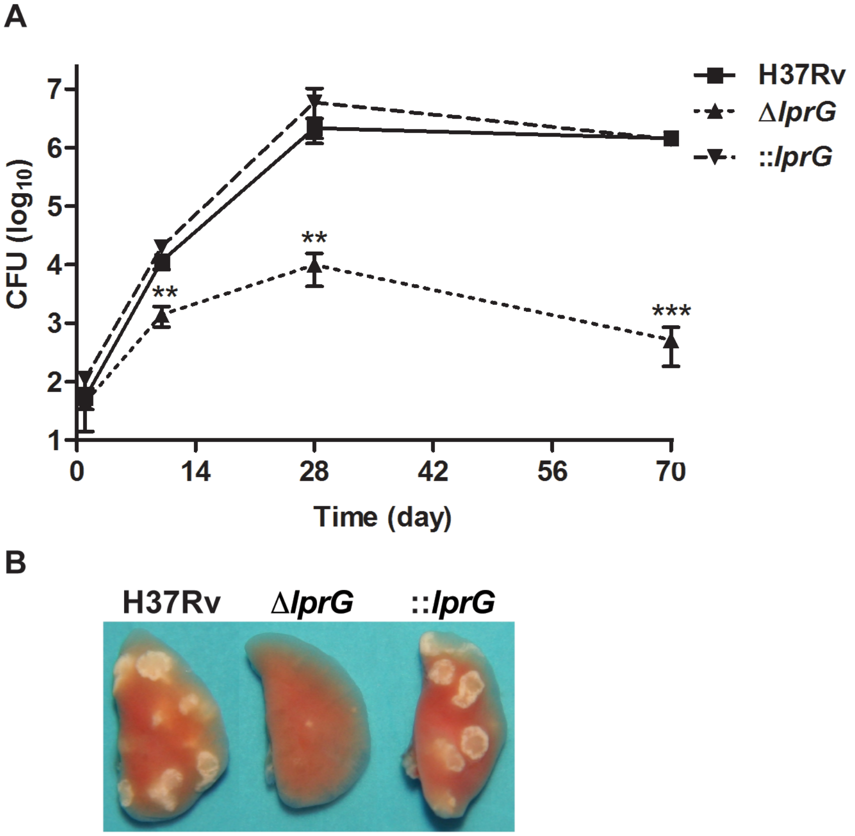

Figure 6. The *lprG* mutant cannot persist in mouse lung. (A) C57BL/6 mice were infected wild-type (H37Rv), *lprG* mutant (Δ*lprG*), and Δ*lprG* complemented with *lprG*-Rv1410c (::*lprG*) through the aerosol route and bacterial load in the lung was measured at the indicated time points. Data points show average CFU count ±SD of 4 mice per time point. (B) Representative lungs on day 70 post-infection. Data is representative of two independent experiments. Δ*lprG* and ::*lprG* were compared to H37Rv. **P<0.01; ***P<0.005.
doi:10.1371/journal.ppat.1004376.g006

and colleagues showed that under non-perturbing conditions mycobacteria express a thick capsule that labels with anti-LAM but under perturbing conditions mycobacteria shed their capsule [6]. Although anti-LAM surface labeling was reduced under perturbing conditions, as much as 80% of mycobacteria in some experiments stained positive with anti-lipoglycan antiserum. Thus, although we were not able to compare surface LAM properties of the *lprG* mutant to wild-type bacteria under non-perturbing conditions due to bacterial clumping, our surface LAM staining results in wild-type bacteria are consistent with the above study; perturbing conditions still allowed us to detect a measurable difference in the *lprG* mutant. Furthermore, although the biological significance of perturbing and non-perturbing conditions in the context of lung environments is unknown, the fact that cell surface alteration has been reported under non-perturbing conditions [21,30] suggests the *lprG* mutant has a surface defect that manifests itself under both perturbing and non-perturbing conditions.

The findings of this study have translational implications for tuberculosis control and eradication. First, the elimination of the *lprG* mutant from mouse lung during the adaptive phase of infection makes LprG and P55 attractive drug targets. There has been a growing interest in repurposing of efflux pump inhibitors currently approved for other disorders as a way of increasing the susceptibility of *M. tuberculosis* to anti-tuberculosis drugs and thus shortening the duration of therapy [34,35]. Using the cell-imprinting assay described in this study, we may be able to identify efflux pump inhibitors that target P55 and chemically achieve an LprG null phenotype. The repurposing of efflux pump inhibitors with activity against P55 may be particularly useful for treatment of strains that are resistant to existing drugs. Secondly, the elimination of the *lprG* mutant from the mouse lung with the onset of adaptive immunity together with the loss of immunomodulatory effects of surface LAM in the *lprG* mutant raise the possibility that this mutant elicits an adaptive immune response that is capable of achieving sterilizing immunity. Further studies are underway to determine whether elimination of the *lprG* mutant is due to a more able adaptive immune response or due to a less resistant mutant. If the former is true, the *lprG* mutant may have utility as a vaccine candidate for prevention of *M. tuberculosis* infection.

Materials and Methods

Ethics statement

All animal experiments were done in accordance with procedures approved by the NYU School of Medicine Institutional Animal Care and Use Committee and in strict accordance with the recommendations in the Guide for the Care and Use of Laboratory Animals of the National Institutes of Health under the Assurance of Compliance Number A3435-01.

Bacteriologic media

M. tuberculosis was grown in Middlebrook 7H9 broth (DifCo) supplemented with 0.2% glycerol, 10% OADC (DifCo), and

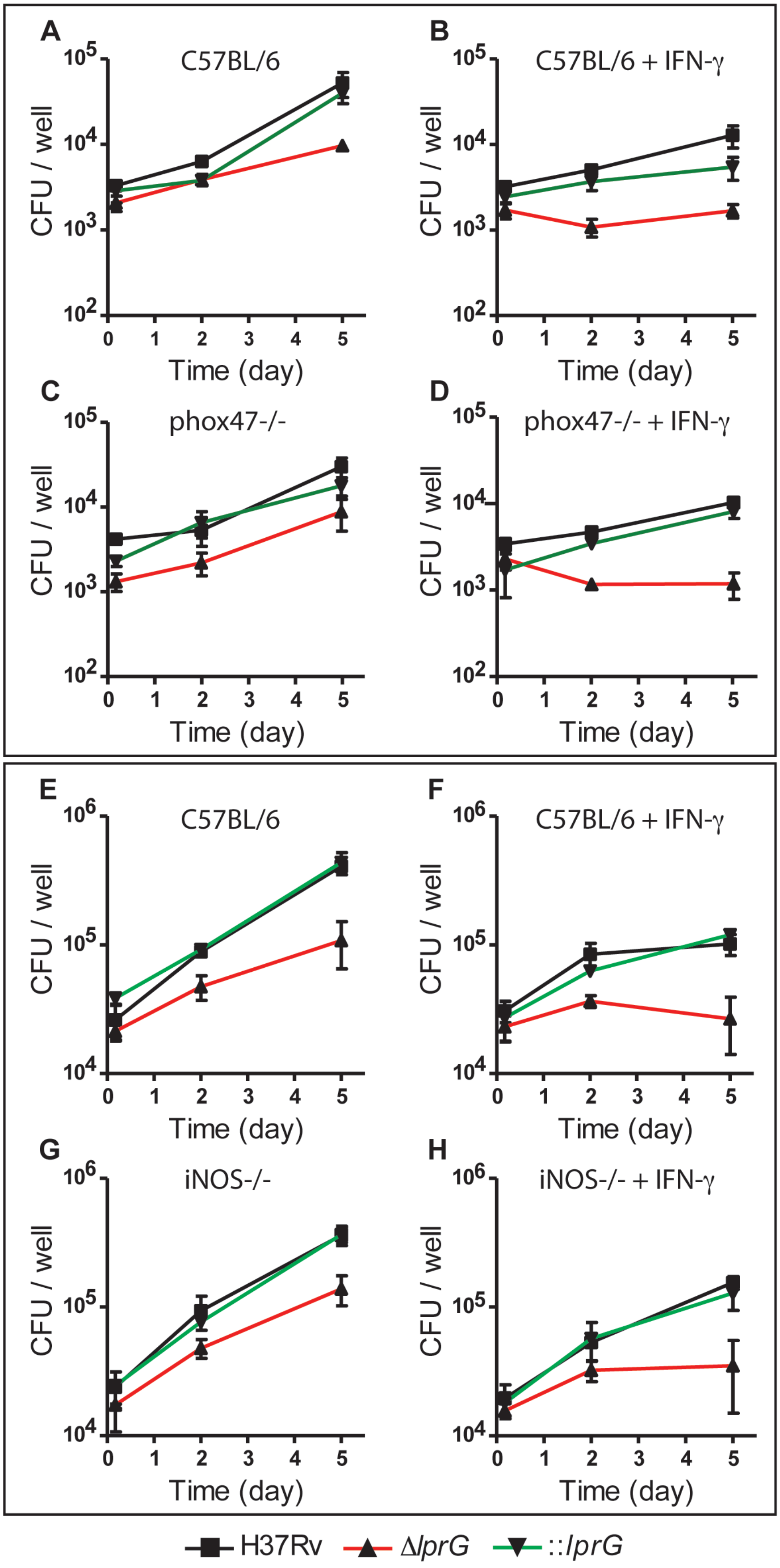
A
C57BL/6
B
C57BL/6 + IFN-γ
C
phox47-/-
D
phox47-/- + IFN-γ
E
C57BL/6
F
C57BL/6 + IFN-γ
G
iNOS-/-
H
iNOS-/- + IFN-γ
CFU / well
Time (day)
H37Rv
ΔlprG
::lprG

Figure 7. The *lprG* mutant is attenuated in mouse macrophages. Resting and IFN-γ-activated BMMΦ from C57BL/6 (A, B, E, and F), p-47phox−/− (C and D), and iNOS−/− (G, H) mice were infected with wild-type (H37Rv), *lprG* mutant (Δ*lprG*), and Δ*lprG* complemented with *lprG*-Rv1410c (::*lprG*) at MOI of 1:10 (A–D) and 1:40 (E–H). Intracellular bacteria were enumerated on Middlebrook 7H9 agar on the indicated days. Data points represent average CFU ±SD of triplicate wells. Data is representative of two to three independent experiments.
doi:10.1371/journal.ppat.1004376.g007

0.05% Tween-80. For lipid and lipoglycan analysis bacteria were grown in Glycerol-Alanine-Salts (GAS) medium with shaking. Middlebrook 7H9 agar with 0.2% glycerol, 10% ADC was used for enumeration of bacterial colonies.

Strain construction

The *lprG* deletion mutant (Δ*lprG*) was constructed in the H37Rv strain with conditionally replicating mycobacteriophages as previously described [36]. Primer sequences used in this study are shown in Table S5. To complement the Δ*lprG*, two PCR-amplified fragments of 1571 bp and 1060 bp, encoding the *lprG*-Rv1410c operon and the native promoter, were directionally cloned into the integrative plasmid pMV306. The Δ*lprG* and the Δ*lprG* complemented with Δ*lprG*-Rv1410c (::*lprG*) were confirmed by real-time PCR using genomic DNA and cDNA, respectively (Figure S1). Construction of Δ*lspA* and Δ*whiB3* was previously described [37,38].

Preparation and analysis of lipids and lipoglycans

Capsular materials were extracted with glass beads as described previously [24]. Culture filtrates (0.22 µm filters) containing the secreted and shed material were similarly prepared. Capsular and culture filtrate materials were concentrated and dialyzed extensively against water prior to analyses. Lipoglycans were prepared from bacterial cells and analyzed by SDS-PAGE on 10–20% gradient Tricine SDS-polyacrylamide gels (Invitrogen) as described previously [39]. Lipids were extracted from bacterial cells, capsular materials and culture filtrates as previously described [40] and analyzed by TLC on aluminum-backed silica gel 60-precoated plates F_{254} (E. Merck) in a variety of solvent systems [41]. The phosphatidylinositol mannoside content of the cells was also examined by submitting total lipids to MALDI-TOF MS analysis [39]. Mycolic acid methyl esters from delipidated cells were prepared as described earlier [40] and analyzed by TLC using *n*-hexane/ethyl acetate (95:5) as the eluent and LC-MS [42]. The glycosyl composition of whole delipidated cells, culture filtrates and capsular materials were determined by acid hydrolysis with 2 M trifluoroacetic acid followed by analysis of the alditol acetate sugar derivatives by GC-MS as previously described [43]. Capsular α-glucan content was measured with a spot immunoblot assay. Blots were blocked and incubated with goat anti-phosphatidylinositol-glycans polyclonal IgG antibody (Santa Cruz Biotechnology), at a dilution of 1/200, and donkey anti-goat IgG-HRP secondary antibody, at a dilution of 1/200. The blot was washed with TBS-0.5% Tween-20, and then treated with 30% 3,3′-diaminobenzidine tetrahydrochloride solution plus 0.0005% H_2O_2 until dots became visible. Dead-dextran (Sigma) and glycogen (Santa Cruz Biotechnology) were used as positive controls.

LAM immunoblot

Whole cell lysates were prepared as previously described [27]. Soluble protein concentration was measured (BCA Protein Assay; Pierce Biotechnology). Lysates were normalized to protein content and separated on a 15% SDS/PAGE gel and transferred to a PVDF membrane. The membrane was blocked with 3% BSA in TBS-0.5% Tween-20, and then incubated with rabbit anti-LAM polyclonal IgG (α-LAM; BEI Resources), at a dilution of 1/200, overnight at 4°C. The membrane was washed with TBS-0.5% Tween-20 and incubated with goat anti-rabbit IgG-HRP (Santa Cruz Biotechnology), at a dilution of 1/400, for 90 min at 24°C. The membranes and images were developed as described above.

Surface LAM staining

Mid-log shaking cultures were centrifuged at 800 rpm for 8 min to remove clumps. Bacilli were washed with PBS and fixed in 4% paraformaldehyde, and then resuspended in PBS and incubated in 5% bovine serum albumin for 1 h. Bacilli were sedimented and suspended in 3% bovine serum albumin with α-LAM, at a dilution of 1/100, or mouse anti-LAM monoclonal IgG (CS-35; BEI Resources), at a dilution of 1/150, and incubated overnight at 4°C. Bacilli were washed with PBS-0.02% Tween-20 and resuspended in PBS with goat anti-rabbit IgG Fab2-Alexa fluor (Cell Signalling), at a dilution of 1/200, and goat anti-mouse IgG IgG-Dylight (Cell Signalling), at a dilution of 1/100, respectively, for 2 h at 24°C. Bacilli were washed, mounted with VECTA-SHIELD HardSet (Vector Laboratory), and visualized using a fluorescent microscope. The reader was blinded to the identity of each sample and graded each bacillus as positive (dim and bright) or negative staining.

Cell-imprinting

Mid-log shaking cultures were sedimented, washed in PBS, and then fixed in 4% paraformaldehyde. Cell-imprinting was performed as previously described [25,26]. Briefly, one drop of bacteria in PBS at an OD_{580} of 2 was placed on a polystyrene slide (Evergreen) and incubated overnight at 4°C. The excess liquid on the slide was removed by spinning the slide at 2000 rpm for 30 sec. The slide was air-dried, heated at 60°C for 2 h, rinsed with deionized water, air-dried, and used as a template for imprinting.

To create a cell imprint, PDMS curing mixture (monomer to cross-linker ratio 10:1) (GE Silicone) with cyclohexane (2:1 volume) and 0.5% poly (ethylene glycol) methyl ether methacrylate (Sigma Aldrich) was spin-coated onto a microscope slide for 30 sec at 1500 rpm. The PDMS was pre-cured at 80°C for 12 min. The template stamp was pressed into PDMS and incubated at 37°C for 8 h, followed by 60°C for 1 h. The template was peeled off and the imprinted polymer film was sonicated in distilled water for 5 min. The cell-imprinted substrates were then inspected with a scanning probe microscope (XE-70, Park Systems) under non-contact AFM (tapping) mode, using etched silicon cantilevers (resonance frequency ~300 kHz, tip radius <10 nm) with medium-low tip oscillation damping ~15% and a scan rate of 0.2 Hz. Geometric information of the imprints was extracted using XEI (Park Systems).

For cell capture studies, bacterial suspensions in PBS at an OD_{A580} of 0.01 were incubated in PBS alone and with α-LAM at a dilution of 1/20, with CS-35 at a dilution of 1/10, rabbit IgG isotype control at a dilution of 1/10, and with mouse anti-Ag85 complex monoclonal IgM (CS-90; BEI Resources) at a dilution of 1/10. Bacilli were fluorescently labeled with propidium iodide (Invitrogen) and 25 µl was passed at 5 µl/min through a microfluidic device containing the imprints of H37Rv, Δ*lprG*, and ::*lprG*. After the infusion process, cells captured on each

imprint were imaged using an iXon+ electron-multiplying CCD camera (Andor Technology) and Andor Solis microscopy imaging software. A custom program written on LabVIEW (National Instruments) was used to control the stage to allow stepwise scanning of 8 adjacent view fields (280 μm×280 μm). The images were analyzed using ImageJ (National Institutes of Health) and the average number of cells captured per 8 view fields was measured. The operator was not blinded to where the scanning was initiated or scoring of images, however, blinded scoring of H37Rv, Δ*lprG*, and ::*lprG* sample images from Figure 2C by a blinded reader showed both quantitative and statistical agreement with the unblinded results (interclass correlation coefficient of 0.9).

SEM

Single cell suspensions of bacilli were fixed overnight at 24°C on poly-lysine coated glass slides in 0.1 M sodium cacodylate pH 7.3 containing 4% formaldehyde and 2% glutaraldehyde, followed by 1% aqueous osmium tetroxide for 1 h, and then dehydrated in an increasing ethanol concentrations series (50% to 100%), and finally critical-point-dried in liquid carbon dioxide using Tousimis Autosamdri 814. Immediately after the process, the samples were coated with a thin layer (7.5 nm thick) Au/Pt mixture using Denton DeskII sputtering system, and inspected using field emission SEM (Sigma HD, Zeiss) under high vacuum mode with working distance of 5 mm. Cellular measurements were performed using Freehand software (Adobe).

Phagosome-Lysosome co-localization

RAW 264.7 cells were cultivated in Dulbecco's Modified Eagle Medium (Corning) supplemented with 10% fetal bovine serum, 1% L-glutamine, and 1% non-essential amino acids and plated on Lab-Tek chamber slides. Lysosomal and bacterial staining were performed as previously described [44,45]. Briefly, RAW cells were treated with 50 nM LysoTracker-Red DND-99 (Life Technology) with and without MMR blocking with 4 mg/ml mannan (Sigma) or 0.5 μg/ml anti-MMR/CD206 pAb (R&D Systems). Cells were incubated for 30 min, and then infected with FITC-labeled bacteria at MOI of 1:100 for 2 h. Cells were washed with PBS, fixed in 4% paraformaldehyde, mounted with VECTASHIELD HardSet, and visualized using a fluorescent microscope. The reader was blinded to the identity of each sample.

Macrophage infections

Bone marrow-derived macrophages (BMMΦ) were isolated from mice as previously described [38] and plated at a density of 5×10^5 cells per well. Bacterial cultures were sedimented, washed with PBS, and resuspended in BMMΦ media. Monolayers were infected at MOI of 1:10 or 1:40 and activated with recombinant IFN-γ 20 ng/ml. At 4 h post-infection, monolayers were washed with PBS and replaced in fresh BMMΦ media. At the indicated times, monolayers were lysed in 1 ml of PBS with 0.5% triton X-100 and bacterial counts were enumerated.

Mouse infections

C57BL/6 mice were purchased from The Jackson Laboratory and housed under specific pathogen-free conditions. Mice were used in compliance with NYU institutional policies. Aerosol infections were performed in an inhalation exposure device (Glas-Col) as previously described [38]. On designated dates, mice were sacrificed and the lungs were homogenized in 2 ml of PBS with 0.5% tween 80 and the bacterial CFU counts were enumerated on Middlebrook 7H11.

Statistical analysis

The student t-test was used to determine significant differences between groups.

Supporting Information

Figure S1 Construction of the *lprG* mutant. (A) The genomic map of *lprG*-Rv1410c operon in *M. tuberculosis* H37Rv. (B) The allelic exchange substrate used to construct the *lprG* mutant (Δ*lprG*). Hyg, hygromycin. (C) The genomic map of *lprG*-Rv1410c operon in Δ*lprG*. The black box represents region deleted from *lprG*. (D) The *lprG*-Rv1410c genomic fragment used to complement Δ*lprG*. (E) Real-time PCR amplification plot showing amplification of *lprG* from genomic DNA. (F) Reverse-transcriptase real-time PCR amplification plot showing amplification of Rv1410c from cDNA.
(TIF)

Figure S2 The *lprG* mutant has normal growth kinetics in broth. Mid-log shaking broth cultures of wild-type (H37Rv), *lprG* mutant (Δ*lprG*), and Δ*lprG* complemented with *lprG*-Rv1410c (::*lprG*) were diluted to OD_{A580} of 0.05 in Middlebrook 7H9 broth with Tween-80. (A) The optical density of shaking cultures was measured daily. (B) Images of colonies grown on Middlebrook 7H9 agar for 3 wk. White bars mark the borders of colonies.
(TIF)

Figure S3 The *lprG* mutant has normal cellular dimensions. SEM micrographs of H37Rv (A and B) and Δ*lprG* (C and D). Wild-type (H37Rv) and *lprG* mutant (Δ*lprG*) bacilli grown in Middlebrook 7H9 broth were fixed with 4% formaldehyde, 2% glutaraldehyde, followed by 1% aqueous osmium tetroxide, and then dehydrated and coated with a thin layer of Au/Pt mixture before inspection using SEM. AFM images of imprints of H37Rv (E) and Δ*lprG* (F) in PDMS polymer. Pre-fixed bacilli were dried on polystyrene glass slides and used as template stamps to press into pre-cured PDMS polymer. The polymer was cured, the stamp was peeled off, and then the geometrical shape of the imprints was extracted using AFM. (G) Dimensions of H37Rv and Δ*lprG* obtained with SEM and AFM. Each value is an average of nine bacilli ±SD.
(TIF)

Figure S4 Phosphatidylinositol mannoside and trehalose dimycolate content of the *lprG* mutant. Thin-layer chromatograms of total lipids extracted from wild-type (H37Rv; 1), *lprG* mutant (Δ*lprG*; 2), and Δ*lprG* complemented with *lprG*-Rv1410c (::*lprG*; 3). The same amounts of total lipids were loaded for each strain. TLC plates were run in the solvent system $CHCl_3$/CH_3OH/H_2O (20:4:0.5, by vol.) and revealed with α-naphthol. TDM, trehalose dimycolates; SL, sulfolipid; CL, cardiolipin; PIM_2, phosphatidylinositol dimannosides; TDM Std, TDM standard.
(TIF)

Figure S5 Cell-imprinting assay using bacteria pretreated with an isotype control. (A) Samples of wild-type (H37Rv), *lprG* mutant (Δ*lprG*), and Δ*lprG* complemented with *lprG*-Rv1410c (::*lprG*) suspended in PBS at an OD_{A580} of 0.01 were pretreated with rabbit IgG isotype control at a dilution of 1/10 and then fluorescently labeled with propidium iodide and 25 μl was flowed at 5 μl/min through a microfluidic device containing the imprints of H37Rv, Δ*lprG*, and ::*lprG*. The average number of captured bacilli per eight view fields was measured using a fluorescent microscope. Data is representative of three independent

experiments. Capture on imprints of Δ*lprG* and ::*lprG* was compared to H37Rv. *P<0.05; **P<0.01.
(TIF)

Table S1 Number of H37Rv, Δ*lprG*, and ::*lprG* captured on cell-imprints of H37Rv, Δ*lprG*, and ::*lprG*.
(DOC)

Table S2 Number of H37Rv, Δ*lprG*, and ::*lprG* captured on cell-imprints of H37Rv, Δ*lprG*, and ::*lprG* after pre-incubation of samples with anti-LAM polyclonal antibody α-LAM.
(DOC)

Table S3 Number of H37Rv, Δ*lprG*, and ::*lprG* captured on cell-imprints of H37Rv, Δ*lprG*, and ::*lprG* after pre-incubation of samples with anti-Ag85b complex monoclonal antibody CS-90.
(DOC)

Table S4 Number of H37Rv, Δ*lprG*, and ::*lprG* captured on cell-imprints of H37Rv, Δ*lprG*, and ::*lprG* after pre-incubation of samples with anti-LAM isotype control antibody.
(DOC)

Table S5 Primers used in this study.
(PDF)

Acknowledgments

We thank Jeffery Glenn for providing reagents, Manuel Amieva for assistance with imaging, and Christopher Becker for conducting mass spectrometry.

Author Contributions

Conceived and designed the experiments: RLG KR AB MJ NB. Performed the experiments: RLG KR AB SB SG NB. Analyzed the data: RLG KR AB MG NB. Contributed reagents/materials/analysis tools: RNZ. Contributed to the writing of the manuscript: RLG KR MJ SE JDE NB.

References

1. World Health Organization. Global tuberculosis report 2013. Available: http://apps.who.int/iris/bitstream/10665/91355/1/9789241564656_eng.pdf?ua = 1 Accessed 10MMarch2014.
2. Philips JA, Ernst JD. (2012) Tuberculosis pathogenesis and immunity. Annu Rev Pathol 7: 353–384.
3. Smith I. (2003) *Mycobacterium tuberculosis* pathogenesis and molecular determinants of virulence. Clin Microbiol Rev 16: 463–496.
4. Brennan PJ, Nikaido H. (1995) The envelope of mycobacteria. Annu Rev Biochem 64: 29–63.
5. Jarlier V, Nikaido H. (1994) Mycobacterial cell wall: Structure and role in natural resistance to antibiotics. FEMS Microbiol Lett 123: 11–18.
6. Sani M, Houben EN, Geurtsen J, Pierson J, de Punder K, et al. (2010) Direct visualization by cryo-EM of the mycobacterial capsular layer: A labile structure containing ESX-1-secreted proteins. PLoS Pathog 6: e1000794.
7. Pitarque S, Larrouy-Maumus G, Payre B, Jackson M, Puzo G, et al. (2008) The immunomodulatory lipoglycans, lipoarabinomannan and lipomannan, are exposed at the mycobacterial cell surface. Tuberculosis 88: 560–565.
8. Mishra AK, Driessen NN, Appelmelk BJ, Besra GS. (2011) Lipoarabinomannan and related glycoconjugates: Structure, biogenesis and role in *Mycobacterium tuberculosis* physiology and host-pathogen interaction. FEMS Microbiol Rev 35: 1126–1157.
9. Briken V, Porcelli SA, Besra GS, Kremer L. (2004) Mycobacterial lipoarabinomannan and related lipoglycans: From biogenesis to modulation of the immune response. Mol Microbiol 53: 391–403.
10. Hunter SW, Brennan PJ. (1990) Evidence for the presence of a phosphatidylinositol anchor on the lipoarabinomannan and lipomannan of *Mycobacterium tuberculosis*. J Biol Chem 265: 9272–9279.
11. Strohmeier GR, Fenton MJ. (1999) Roles of lipoarabinomannan in the pathogenesis of tuberculosis. Microbes Infect 1: 709–717.
12. Chua J, Vergne I, Master S, Deretic V. (2004) A tale of two lipids: *Mycobacterium tuberculosis* phagosome maturation arrest. Curr Opin Microbiol 7: 71–77.
13. Hmama Z, Sendide K, Talal A, Garcia R, Dobos K, et al. (2004) Quantitative analysis of phagolysosome fusion in intact cells: Inhibition by mycobacterial lipoarabinomannan and rescue by an 1α,25-dihydroxyvitamin D3-phosphoinositide 3-kinase pathway. J Cell Sci 117: 2131–2140.
14. Kang PB, Azad AK, Torrelles JB, Kaufman TM, Beharka A, et al. (2005) The human macrophage mannose receptor directs *Mycobacterium tuberculosis* lipoarabinomannan-mediated phagosome biogenesis. J Exp Med 202: 987–999.
15. Schlesinger LS. (1993) Macrophage phagocytosis of virulent but not attenuated strains of *Mycobacterium tuberculosis* is mediated by mannose receptors in addition to complement receptors. J Immunol 150: 2920–2930.
16. Villeneuve C, Gilleron M, Maridonneau-Parini I, Daffe M, Astarie-Dequeker C, et al. (2005) Mycobacteria use their surface-exposed glycolipids to infect human macrophages through a receptor-dependent process. J Lipid Res 46: 475–483.
17. Sutcliffe IC, Russell RR. (1995) Lipoproteins of gram-positive bacteria. J Bacteriol 177: 1123–1128.
18. Drage MG, Tsai HC, Pecora ND, Cheng TY, Arida AR, et al. (2010) *Mycobacterium tuberculosis* lipoprotein LprG (Rv1411c) binds triacylated glycolipid agonists of toll-like receptor 2. Nat Struct Mol Biol 17: 1088–1095.
19. Cole ST, Brosch R, Parkhill J, Garnier T, Churcher C, et al. (1998) Deciphering the biology of *Mycobacterium tuberculosis* from the complete genome sequence. Nature 393: 537–544.
20. Bianco MV, Blanco FC, Imperiale B, Forrellad MA, Rocha RV, et al. (2011) Role of P27–P55 operon from *Mycobacterium tuberculosis* in the resistance to toxic compounds. BMC Infect Dis 11: 1–9.
21. Farrow MF, Rubin EJ. (2008) Function of a mycobacterial major facilitator superfamily pump requires a membrane-associated lipoprotein. J Bacteriol 190: 1783–1791.
22. Sassetti CM, Rubin EJ. (2003) Genetic requirements for mycobacterial survival during infection. Proc Natl Acad Sci U S A 100: 12989–12994.
23. Bigi F, Gioffre A, Klepp L, Santangelo MP, Alito A, et al. (2004) The knockout of the lprG-Rv1410 operon produces strong attenuation of *Mycobacterium tuberculosis*. Microbes Infect 6: 182–187.
24. Ortalo-Magne A, Dupont MA, Lemassu A, Andersen AB, Gounon P, et al. (1995) Molecular composition of the outermost capsular material of the tubercle bacillus. Microbiology 141: 1609–1620.
25. Schirhagl R, Hall EW, Fuereder I, Zare RN. (2012) Separation of bacteria with imprinted polymeric films. Analyst 137: 1495–1499.
26. Ren K, Zare RN. (2012) Chemical recognition in cell-imprinted polymers. ACS Nano 6: 4314–4318.
27. Banaei N, Kincaid EZ, Lin SY, Desmond E, Jacobs WR, Jr, et al. (2009) Lipoprotein processing is essential for resistance of *Mycobacterium tuberculosis* to malachite green. Antimicrob Agents Chemother 53: 3799–3802.
28. Singh A, Crossman DK, Mai D, Guidry L, Voskuil MI, et al. (2009) *Mycobacterium tuberculosis* WhiB3 maintains redox homeostasis by regulating virulence lipid anabolism to modulate macrophage response. PLoS Pathog 5: e1000545.
29. Desvignes L, Ernst JD. (2009) Interferon-γ-responsive nonhematopoietic cells regulate the immune response to *Mycobacterium tuberculosis*. Immunity 31: 974–985.
30. Ramon-Garcia S, Martin C, Thompson CJ, Ainsa JA. (2009) Role of the *Mycobacterium tuberculosis* P55 efflux pump in intrinsic drug resistance, oxidative stress responses, and growth. Antimicrob Agents Chemother 53: 3675–3682.
31. Chan J, Fan XD, Hunter SW, Brennan PJ, Bloom BR. (1991) Lipoarabinomannan, a possible virulence factor involved in persistence of *Mycobacterium tuberculosis* within macrophages. Infect Immun 59: 1755–1761.
32. Garg A, Barnes PF, Roy S, Quiroga MF, Wu S, et al. (2008) Mannose-capped lipoarabinomannan- and prostaglandin E2-dependent expansion of regulatory T cells in human *Mycobacterium tuberculosis* infection. Eur J Immunol 38: 459–469.
33. Ehlers S. (2010) DC-SIGN and mannosylated surface structures of *Mycobacterium tuberculosis*: A deceptive liaison. Eur J Cell Biol 89: 95–101.
34. Adams KN, Takaki K, Connolly LE, Wiedenhoft H, Winglee K, et al. (2011) Drug tolerance in replicating mycobacteria mediated by a macrophage-induced efflux mechanism. Cell 145: 39–53.

35. Gupta S, Tyagi S, Almeida DV, Maiga MC, Ammerman NC, et al. (2013) Acceleration of tuberculosis treatment by adjunctive therapy with verapamil as an efflux inhibitor. Am J Respir Crit Care Med 188: 600–607.
36. Bardarov S, Bardarov Jr S, Jr, Pavelka Jr MS, Jr, Sambandamurthy V, Larsen M, et al. (2002) Specialized transduction: An efficient method for generating marked and unmarked targeted gene disruptions in *Mycobacterium tuberculosis*, M. bovis BCG and M. smegmatis. Microbiology 148: 3007–3017.
37. Banaiee N, Jacobs WR, Jr, Ernst JD. (2006) Regulation of *Mycobacterium tuberculosis whiB3* in the mouse lung and macrophages. Infect Immun 74: 6449–6457.
38. Banaiee N, Kincaid EZ, Buchwald U, Jacobs WR, Jr, Ernst JD. (2006) Potent inhibition of macrophage responses to IFN-γ by live virulent *Mycobacterium tuberculosis* is independent of mature mycobacterial lipoproteins but dependent on TLR2. J Immunol 176: 3019–3027.
39. Kaur D, Berg S, Dinadayala P, Gicquel B, Chatterjee D, et al. (2006) Biosynthesis of mycobacterial lipoarabinomannan: Role of a branching mannosyltransferase. Proc Natl Acad Sci U S A 103: 13664–13669.
40. Stadthagen G, Kordulakova J, Griffin R, Constant P, Bottova I, et al. (2005) p-hydroxybenzoic acid synthesis in *Mycobacterium tuberculosis*. J Biol Chem 280: 40699–40706.
41. Rousseau C, Sirakova TD, Dubey VS, Bordat Y, Kolattukudy PE, et al. (2003) Virulence attenuation of two mas-like polyketide synthase mutants of *Mycobacterium tuberculosis*. Microbiology 149: 1837–1847.
42. Shui G, Bendt AK, Pethe K, Dick T, Wenk MR. (2007) Sensitive profiling of chemically diverse bioactive lipids. J Lipid Res 48: 1976–1984.
43. McNeil M, Chatterjee D, Hunter SW, Brennan PJ. (1989) Mycobacterial glycolipids: Isolation, structures, antigenicity, and synthesis of neoantigens. Methods Enzymol 179: 215–242.
44. N'Diaye EN, Darzacq X, Astarie-Dequeker C, Daffe M, Calafat J, et al. (1998) Fusion of azurophil granules with phagosomes and activation of the tyrosine kinase hck are specifically inhibited during phagocytosis of mycobacteria by human neutrophils. J Immunol 161: 4983–4991.
45. Desai SD, Reed RE, Babu S, Lorio EA. (2013) ISG15 deregulates autophagy in genotoxin-treated ataxia telangiectasia cells. J Biol Chem 288: 2388–2402.
