# Supplementary figures and images for: Correction: LprG-Mediated Surface Expression of Lipoarabinomannan Is Essential for Virulence of Mycobacterium tuberculosis
Source: PLoS Pathog. 2015 Dec 9;11(12):e1005336. doi: 10.1371/journal.ppat.1005336 (PMC4674111; doi:10.1371/journal.ppat.1005336)

## Slide 1
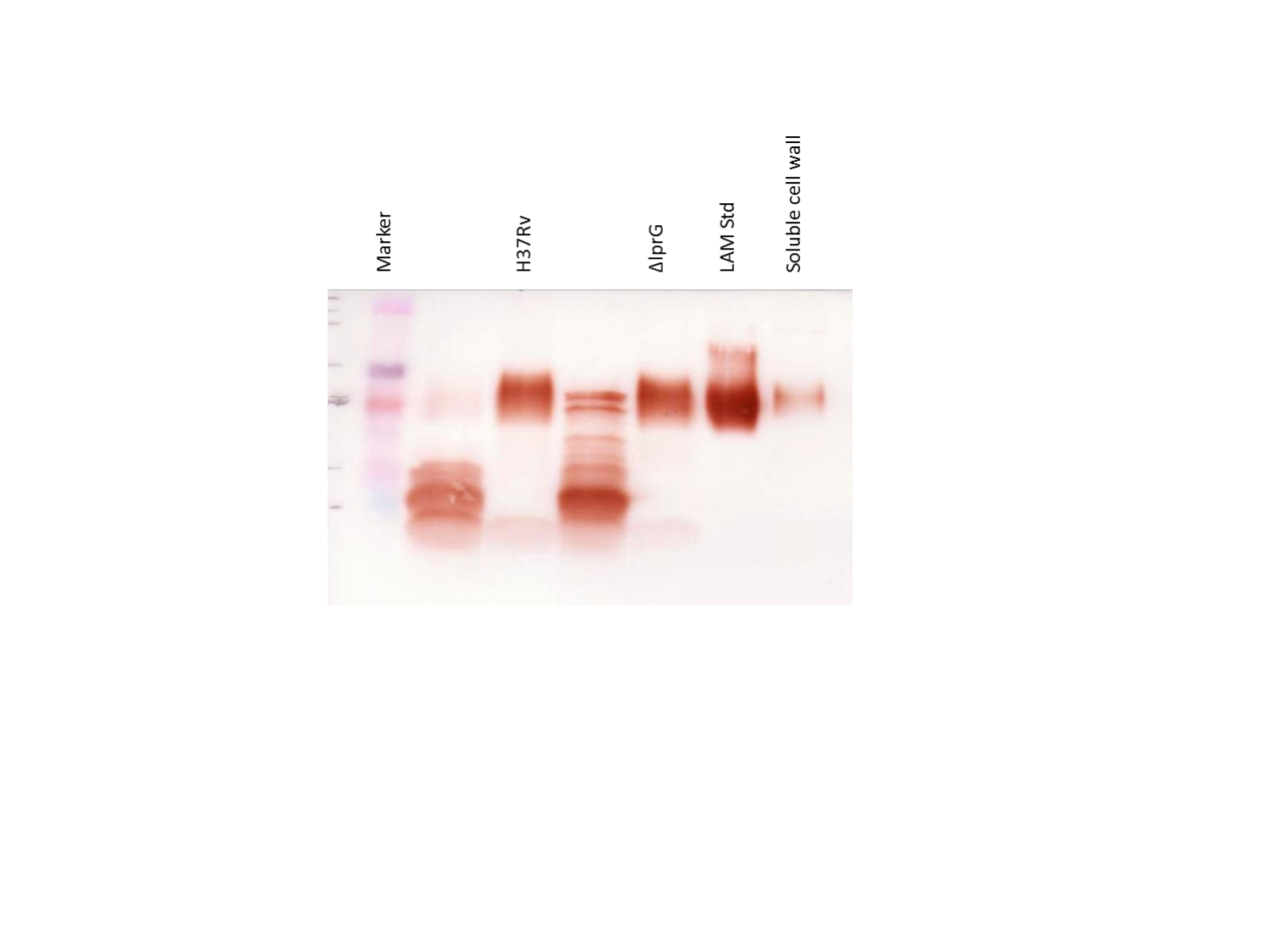

Supplement: S1 Fig — Extracts normalized to protein concentration were separated on a 15% SDS/PAGE gel and transferred to PVDF membrane. The blot was blocked, and then stained with anti-LAM pAb (α-LAM) followed by goat anti-rabbit IgG-HRP secondary antibody. The blot was washed and imaged after adding 30% 3,3’-diaminobenzidine tetrahydrochloride solution plus 0.0005% H2O2. LAM Std, purified H37Rv LAM standard. Soluble cell wall, purified H37Rv cell wall extract. (PPTX) [file ppat.1005336.s001.pptx]
